# Gamma-Tubulin Is Required for Bipolar Spindle Assembly and for Proper Kinetochore Microtubule Attachments during Prometaphase I in *Drosophila* Oocytes

**DOI:** 10.1371/journal.pgen.1002209

**Published:** 2011-08-11

**Authors:** Stacie E. Hughes, J. Scott Beeler, Angela Seat, Brian D. Slaughter, Jay R. Unruh, Elisabeth Bauerly, Heinrich J. G. Matthies, R. Scott Hawley

**Affiliations:** 1 Stowers Institute for Medical Research, Kansas City, Missouri, United States of America; 2 Vanderbilt University School of Medicine, Nashville, Tennessee, United States of America; 3 The University of Kansas, Overland Park, Kansas, United States of America; 4 Department of Molecular Physiology and Biophysics, Vanderbilt University School of Medicine, Nashville, Tennessee, United States of America; 5 Department of Molecular and Integrative Physiology, University of Kansas Medical Center, Kansas City, Kansas, United States of America; The University of North Carolina at Chapel Hill, United States of America

## Abstract

In many animal species the meiosis I spindle in oocytes is anastral and lacks centrosomes. Previous studies of *Drosophila* oocytes failed to detect the native form of the germline-specific *γ*-tubulin (γTub37C) in meiosis I spindles, and genetic studies have yielded conflicting data regarding the role of γTub37C in the formation of bipolar spindles at meiosis I. Our examination of living and fixed oocytes carrying either a null allele or strong missense mutation in the *γtub37C* gene demonstrates a role for γTub37C in the positioning of the oocyte nucleus during late prophase, as well as in the formation and maintenance of bipolar spindles in *Drosophila* oocytes. Prometaphase I spindles in *γtub37C* mutant oocytes showed wide, non-tapered spindle poles and disrupted positioning. Additionally, chromosomes failed to align properly on the spindle and showed morphological defects. The kinetochores failed to properly co-orient and often lacked proper attachments to the microtubule bundles, suggesting that γTub37C is required to stabilize kinetochore microtubule attachments in anastral spindles. Although spindle bipolarity was sometimes achieved by metaphase I in both *γtub37C* mutants, the resulting chromosome masses displayed highly disrupted chromosome alignment. Therefore, our data conclusively demonstrate a role for γTub37C in both the formation of the anastral meiosis I spindle and in the proper attachment of kinetochore microtubules. Finally, multispectral imaging demonstrates the presences of native γTub37C along the length of wild-type meiosis I spindles.

## Author Summary

Proper chromosome segregation during cell division is essential. Missegregation of mitotic chromosomes leads to cell death or cancer, and chromosome missegregation during meiosis leads to miscarriage and birth defects. Cells utilize a bipolar microtubule-based structure known as the meiotic or mitotic spindle to segregate chromosomes. Because proper bipolar spindle formation is critically important for chromosome segregation, cells have many redundant mechanisms to ensure that this structure is properly formed. In most animal cells, centrosomes containing γ-tubulin protein complexes help organize and shape the bipolar spindle. Since meiosis I spindles in oocytes lack centrosomes, the mechanisms by which a meiotic bipolar spindle is assembled are not fully understood. In *Drosophila* oocytes it was not clear whether γ-tubulin played a role in bipolar spindle assembly or if it was even present on the meiotic spindle. We demonstrate that γ-tubulin plays vital roles in bipolar spindle formation and maintenance, as well as in aligning the chromosomes on the oocyte spindle. Additionally, we show that γ-tubulin is present on the bipolar spindle in *Drosophila* oocytes. More importantly, we demonstrate that γ-tubulin plays a critical role in the formation of the kinetochore microtubules that are required to properly orient chromosomes on the meiotic spindle.

## Introduction

In mitosis and male meiosis in animals, the establishment of spindle bipolarity is mediated by centrosomes that act as microtubule organizing centers (MTOCs). These structures serve to organize and focus the growing microtubules to form a bipolar spindle. γ-Tubulin is a primary component of MTOCs and is required for mitotic spindle assembly in many organisms (reviewed in [1]). However, in most animal species, including *Drosophila melanogaster*, female meiosis is acentrosomal and the mechanisms by which a bipolar spindle is formed during meiosis I have not been fully elucidated.

Despite the absence of centrosomes, a role for γ-tubulin in female meiosis has been implicated in many organisms. Schuh and Ellenberg [2] have presented strong evidence that the spindle in mouse oocytes is formed by the action of a large number of γ-tubulin-containing MTOCs that are self-organized from a cytoplasmic microtubule network. These authors propose that the progressive clustering of MTOCs, along with the action of a kinesin-5 motor protein, facilitates the formation of a bipolar spindle. This mechanism of acentrosomal spindle assembly is fully consistent with mammalian studies of γ-tubulin during meiosis I that show localization of γ-tubulin throughout the meiosis I spindle [3] and with work by Burbank et al. [4] demonstrating the existence of the minus ends of the microtubules throughout the meiosis I spindle.

These observations lead to a model of spindle assembly in which microtubules are initially nucleated in the region around the chromosomes (possibly by γ-tubulin) and then moved poleward. However, despite the evidence in other female meiotic systems for a role of γ-tubulin in meiosis I spindle assembly and function, the role (if any) of γ-tubulin in the formation of the meiosis I spindle in *Drosophila* oocytes has remained highly controversial [3], [5].

*Drosophila* has two genes encoding γ-tubulin: γ*tub37C* and γ*tub23C* [6]. γTub23C is expressed in all somatic tissues once embryos become cellularized [6], [7]. However, in ovaries γTub23C is only expressed in the mitotically dividing germ cells [6]. After meiosis is initiated, γTub37C accumulates rapidly in the oocyte and nurse cells for use during the rapid embryonic cell divisions [6]. In embryos, γTub37C localizes primarily to the centrosomes, but does show some localization over the length of the mitotic spindle and at the midbody [6], [8]. Although γTub37C is present in the female germline, whether it plays a role in spindle formation during meiosis I has been controversial.

Using similar sets of *γtub37C* mutants, different investigators have obtained highly divergent results with respect to the role of γTub37C in the assembly and function of the first meiotic spindle [8], [9]. Wilson and Borisy [9] examined the effects of a number of γ*tub37C* mutants (including a null allele) on female meiosis I and observed some normal-looking bipolar spindles, leading them to conclude that γTub37C was not essential for either microtubule nucleation or the assembly of the female meiotic spindle. Endow and Hallen [10] reached similar conclusions using a weak loss-of-function allele of *γtub37C*. However, Tavosanis et al. [8] observed significant defects in both spindle morphology and chromosome arrangement during meiosis I in *Drosophila* oocytes. Indeed, in the Tavosanis et al. [8] study, ∼80% of oocytes from mothers hemizygous for two null mutations of γ*tub37C* showed abnormal meiotic figures, including chromosomes randomly arranged across the spindle, spindles that were less dense and less uniform then those observed in wild-type oocytes, and spindles that were not focused at the poles [8]. We will show below that these divergent conclusions with respect to the role of γTub37C in spindle assembly were the result of methodological differences in the manner in which oocytes were collected. The data presented here show that γTub37C is indeed required for spindle assembly and function during prometaphase I (the stage primarily studied by Tavosanis et al. [8]) and that the spindle defects are often ameliorated by metaphase I (the stage primarily studied by Wilson and Borisy [9]).

Even the presence of γTub37C in the meiosis I spindle has been highly contentious. Wilson and Borisy [6], Tavosanis et al. [8] and Matthies et al. [11] all failed to detect γTub37C on the meiosis I spindle by indirect immunofluorescence microscopy. The inability to detect γTub37C on the meiosis I spindle leant support to the genetic data suggesting that γTub37C was not required for spindle formation in meiosis I. However, Endow and Hallen [10] have recently demonstrated the localization of an overexpressed and green fluorescent protein (GFP)-tagged version of γTub37C to the microtubules and poles of the meiosis I spindle. Although this observation shows that γTub37C is capable of localizing to the meiosis I spindle when overexpressed, there are numerous examples of proteins that mislocalize when overexpressed [12], [13]. Thus, it remained to be determined whether endogenous γTub37C is a native component of the meiosis I spindle.

To both resolve these controversies and to explore the role(s) of γTub37C in the acentrosomal spindle, we have characterized defects caused by both a novel point mutation in *γtub37C*, γ*tub37C^P162L^*, and a null mutation of γ*tub37C*, γ*tub37C^3^*, during prometaphase I and metaphase I using both fixed and live oocyte methods. We find that both mutations cause spindle and chromosome defects, demonstrating that γTub37C plays important roles in spindle formation, maintenance, and positioning, as well as chromosome alignment and morphology during prometaphase I. Indeed, mutations in *γtub37C* lead to loss of kinetochore biorientation and altered kinetochore microtubule attachments. Finally, using multispectral imaging we detect endogenously expressed γTub37C on the microtubules of the meiosis I spindle, suggesting that γTub37C acts within the meiotic spindle to execute these essential functions.

## Results

### Mutations in γ*tub37C* cause defects in chromosome alignment during prometaphase I of female meiosis

We examined *Drosophila* oocytes carrying either of two alleles of γ*tub37C* (Genbank AY070558.1) to understand its function during meiosis I. The first mutant, γ*tub37C^P162L^*, was isolated in the course of a screen for EMS-induced recessive female sterile mutants. This mutation resulted in a C to T transition at position 834 that results in a P to L change at amino acid 162 in exon 3 of the γ*tub37C* gene (data not shown). We also examined oocytes carrying a previously characterized early-stop mutation (*γtub37C^3^*), that removes the C-terminal 106 amino acids of the 457 amino acid γTub37C protein, over a deletion that removes the entire γ*tub37*C gene (*γtub37C^3^*/*Df*) [9]. The *γtub37C^3^* mutation is a presumed null allele since the truncated protein could not be detected by Western blot [9].

*Drosophila* females were maintained under two different sets of conditions which yield preparations enriched for either prometaphase I or metaphase I oocytes that were established by Gilliland et al. [14]. For prometaphase I-enriched preparations, mated females were held two to three days post-eclosion on wet yeast paste. Metaphase I-enriched preparations females were collected as virgins and held four to five days on yeast paste in the absence of males.

**Figure 1:**
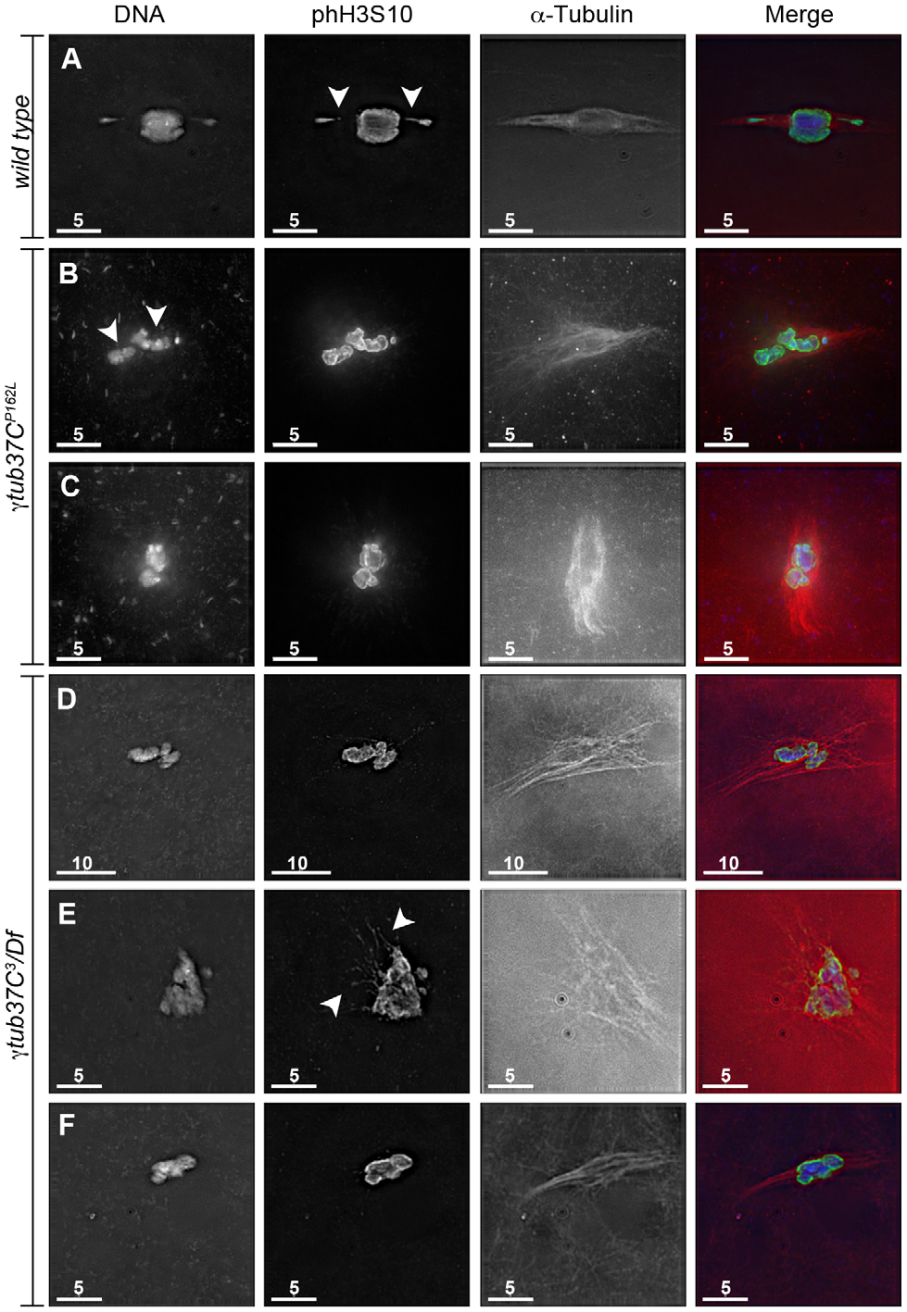
*γtub37C* mutations cause spindle and chromosome defects in oocytes during prometaphase I. Fixed oocytes were treated with antibodies against α-tubulin and histone H3 phosphorylated at serine 10 (phH3S10), as well as the DNA dye DAPI. All oocytes were from prometaphase I-enriched preparations of oocytes. (A) Shows a wild-type oocyte with symmetrical-looking chromosomes and a bipolar spindle. Arrowheads highlight the phH3S10 positive thread projecting from the achiasmate *4^th^* chromosomes. (B) Shows autosomal slippage (arrowheads) on a monopolar spindle in a *γtub37C^P162L^* mutant oocyte. (C) Shows a barrel-like spindle and chromosomes with an abnormal and round morphology in a *γtub37C^P162L^* mutant oocyte. (D) Shows a *γtub37C* ^3^ */Df* mutant oocyte with a barrel-like spindle with abnormal microtubule density and chromosome misalignment. (E) Shows a *γtub37C* ^3^ */Df* mutant oocyte with chromosomes and microtubules that lack clear orientation. Additionally, the phH3S10 antibody highlights threads that project away from the chromosomes rather than connecting achiasmate homologs (arrowheads). (F) Shows a *γtub37C* ^3^ */Df* mutant oocyte with a spindle that is more similar to wild type with clear microtubule directionality and nearly tapered bipolar morphology. The chromosomes are associated and aligned along the spindle axis. (A) and (E) are single Z sections to highlight the phH3S10-labeled threads while (B–D, F) are maximum intensity projections from Z stacks. The scale bars are in microns.

During prometaphase I, in the majority of wild-type oocytes the autosomal bivalents and *X* chromosomes are aligned together at the midzone of the tapered, bipolar spindle with the achiasmate *4^th^* chromosomes either out on the spindle (Figure 1A) or associated with the chiasmate chromosomes (Table 1) [15]. However, upon examination of prometaphase I oocytes from γ*tub37C^P162L^* and *γtub37C^3^/Df* females we observed a wide array of aberrant chromosome configurations (Figure 1B–1E, Figure S1A–S1B, Table 1). As exemplified in Figure 1B, in γ*tub37C^P162L^* mutant oocytes the chiasmate chromosomes often failed to form a single chromosome mass at the spindle midzone, but rather were stretched across the length of the spindle. The most notable feature of Figure 1B is the physical separation of the chiasmate autosomes on the spindle (see arrowheads), a phenomenon that we refer to as autosomal slippage. Autosomal slippage that results in a near total disruption of the overlap between the autosomes on the spindle is not commonly observed in wild-type oocytes [15]. Slippage of the autosomes was observed in 42% of γ*tub37C^P162L^* mutant oocytes and in 23% of γ*tub37C^3^/Df* mutant oocytes (Figure 1B, 1D, Table 1). The lower rate of slippage in the γ*tub37C^3^/Df* mutant oocytes is likely due to the higher rate of severely abnormal chromosome masses in γ*tub37C^3^/Df* mutant oocytes that are described below (Table 1).

**Table 1:** Prometaphase I preparations: DNA configurations.

Genotype	Prometaphase aligned^1^	Metaphase-like^2^	Autosomal slippage^3^	Abnormal chromosome mass^4^	Divided chromosome mass^5^	Abnormal threads^6^	Total
*+/+*	29	11	2	1	0	5	44^7^
*γtub37C^P162L^*	15	4	25	16	0	25	60
*γtub37C* *^3^* */Df*	5	5	13	23	10	39	56

1 Chromosomes are symmetrically arranged on the meiotic spindle, see Figure 1A.

2 Chromosomes have all congressed back to the metaphase plate to form a lemon-shaped configuration, see Figure 5A.

3 Chiasmate chromosomes are randomly arranged on the spindle, see Figure 1B.

4 All the chromosomes are associated but the chromosomal mass is abnormal in structure, see Figure 1E.

5 Chromosomes are not present on the same spindle-like structure, see Figure 5C.

6 Threads staining with DAPI and an antibody to histone H3 phosphorylated at serine 10 are present that do not connect chromosomes.

7 One wild-type oocyte was caught in spindle assembly and not included in any column.

We also observed severe defects in chromosome morphology in mutant prometaphase I oocytes. As exemplified in Figure 1C, the chromosomes from the γ*tub37C* mutant oocytes sometimes appeared rounded and misshapen. Additionally, these chromosome masses did not appear to be properly condensed based on the DAPI staining (Figure 1C). The chromosome morphology defects observed in *γtub37C* mutant oocytes was rarely observed in wild-type oocytes.

As exemplified in Figure 1E and Figure S1A–S1B, the chromosomes of some mutant prometaphase I oocytes failed to align in any direction and displayed both abnormal invaginations and projections away from the main chromosome mass (Figure 1E, Figure S1A–S1B). This lack of obvious alignment was observed in 27% of the γ*tub37C^P162L^* and 41% of γ*tub37C^3^/Df* mutant oocytes (Table 1). Chromosome configurations that appeared more similar to wild-type configurations were observed for a few γ*tub37C^P162L^* and γ*tub37C^3^*/*Df* mutant oocytes (Figure 1F, Figure S1C–S1D, Table 1). Finally, in 18% of γ*tub37C^3^/Df* oocytes the chromosomes had separated far enough apart not to be contained within the same set of microtubules (Table 1).

Thus, as was originally noted by Tavasonis et al. [8], chromosomes fail to align properly on the prometaphase I spindle in γ*tub37C* mutant oocytes. These phenotypes suggest that γTub37C is involved in properly aligning and orienting the chromosomes on the prometaphase I spindle. Additionally, γ*tub37C* mutant oocytes show defects in chromosome morphology, which had not been described previously. Based on these defects, γTub37C appears to function in regulating chromosome alignment and morphology during meiosis I.

### Mutations in γ*tub37C* cause the formation of aberrant DNA threads during prometaphase I

Our ability to recognize chromosomes on the prometaphase I spindle was greatly enhanced by the use of an antibody recognizing histone H3 phosphorylated on serine 10 (phH3S10). Histone H3 serine 10 phosphorylation increases during prophase and peaks at metaphase of mitosis and meiosis (reviewed in [16]). The phH3S10 antibody allowed for unambiguous identification of oocyte nuclei that have progressed to at least prometaphase I even in the absence of normal-looking chromosomes (Figure 1B–1E, Figure S1A–S1B).

While using the phH3S10 antibody in control oocytes, we discovered that this antibody also robustly highlighted the DNA threads that connect achiasmate chromosomes [15]. These threads are hypothesized to be involved in the mechanism by which achiasmate chromosomes can reassociate during their dynamic prometaphase I movements on the meiotic spindle and the subsequent congression of the chromosomes to the metaphase plate during metaphase I [15]. Analyzing DNA threads using DAPI alone is difficult. Often chromosomes will show evidence that threads are present, such as spurs on the ends of the chromosomes, but the full-length thread will be below the level of detection [15]. The phH3S10 antibody allowed for the visualization of complete threads connecting achiasmate chromosomes, such as the thread projecting from the achiasmate *4^th^* chromosomes in Figure 1A in a wild-type oocyte and from both *X* and *4^th^* chromosomes in the *FM7/X* oocytes shown in Figure S2.

Using the phH3S10 antibody to examine thread number and morphology in wild-type oocytes, we primarily observed phH3S10 threads connecting well-separated *4^th^* chromosomes or the absence of threads when achiasmate chromosomes were part of the main chromosome mass. phH3S10-staining threads that failed to project toward another achiasmate chromosome were seen in only 11% of wild-type oocytes, and in these cases only one or two very short threads were observed (Table 1). However, in many γ*tub37C* mutant oocytes multiple abnormal threads were observed projecting away from the chromosomes (Figure 1E and Figure S1A–S1B). These aberrant thread-like structures frequently co-localized with α-tubulin (Figure 1E and Figure S1A–S1B). Such aberrant threads were observed in 70% of γ*tub37C^3^/Df* and 42% of *γtub37C^P162L^* mutant oocytes (Figure 1E, Figure S1A–S1B, Table 1) indicating that functional γTub37C is required for normal DNA thread morphology.

### Mutations in γ*tub37C* cause spindle defects during prometaphase I of female meiosis

In wild-type oocytes at prometaphase I the meiotic spindle has two tapered poles and the spindle is approximately the width of the autosomes on the spindle midzone at its widest point (Figure 1A). Tapered, bipolar spindles were observed in 89% of wild-type oocytes (Table 2).

**Table 2:** Prometaphase I preparations: spindle configurations.

Genotype	Bipolar	Monopolar^1^	Barrel-like^2^	Narrow spindle^3^	Microtubule bundles with no directionality^4^	None^5^	Total
*+/+*	39	3	1	0	0	0	44^6^
*γtub37C^P162L^*	18	13	14	3	9	3	60
*γtub37C* *^3^* */Df*	8	12	14	4	18	0	56

1 One pole is focused while the other end of the spindle is unfocused, see Figure 1B.

2 Microtubules are aligned in the same direction but both poles are unfocused, see Figure 1C.

3 Only isolated thick bundles of microtubules appear to be projecting from the chromosome mass, see Figure 5B.

4 Microtubules are not aligned in a single direction, see Figure 1E.

5 Clear microtubules were not associated with the chromosomes despite good antibody penetration.

6 One wild-type oocyte was caught in spindle assembly and not included in any column. Lack of bipolarity in the monopolar and barrel-like oocytes was typically due to oocytes lying in poor focal planes for imaging.

However, when spindle structure was examined in prometaphase I γ*tub37C* mutant oocytes, we observed either abnormal spindles or the absence of a recognizable spindle in 70% of γ*tub37C^P162L^* mutant oocytes and 86% of γ*tub37C^3^*/*Df* mutant oocytes (Table 2). Less than a third of the spindles in γ*tub37C^P162L^* mutant oocytes, and even fewer in γ*tub37C^3^*/Df mutant oocytes, could be classified as tapered and bipolar (Figure 1F, Figure S1C, Table 2), though some of these bipolar spindles showed minor defects in width and microtubule density.

Monopolar spindles were observed in 22% of prometaphase I γ*tub37C^P162L^* mutant oocytes (Figure 1B, Table 2) and 21% of spindles in γ*tub37C^3^*/*Df* mutant oocytes (Table 2). Barrel-like spindles that lacked tapered poles, but still displayed bidirectionality, were observed in 23% of γ*tub37C^P162L^* mutant oocytes and 25% of γ*tub37C^3^*/Df oocytes (Figure 1C–1D, Table 2). The width of the spindle was not constant in these barrel-like spindles. In the image of the γ*tub37C^P162L^* mutant oocyte that is shown in Figure 1C the spindle is approximately the width of the chromosomes, but in some oocytes the barrel-like spindles were narrower than the chromosomes (Figure S1D).

Finally, in 15% of γ*tub37C^P162L^* and 32% of γ*tub37C^3^/Df* mutant oocytes the microtubules displayed no clear directionality and simply projected in all directions (Figure 1E, Figure S1A–S1B, Table 2). Typically this microtubule morphology was also associated with the most abnormal chromosome morphologies. Three γ*tub37C^P162L^* mutant oocytes failed to show any microtubule association with the chromosomes despite robust antibody penetration. The multitude of aberrant spindle structures suggests that γTub37C is required for both formation of a proper prometaphase I spindle and for maintenance of a tapered bipolar spindle.

### Mutations in γ*tub37C* disrupt kinetochore orientation and kinetochore-microtubule attachments during prometaphase I

**Figure 2:**
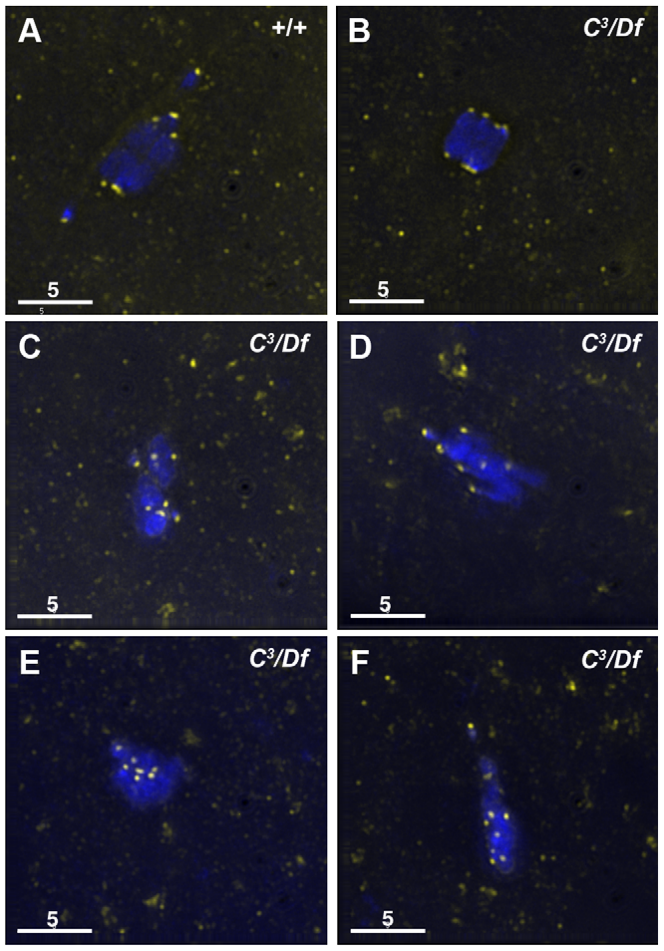
Kinetochores fail to properly biorient during prometaphase I in *γtub37C* ^3^ */Df* mutant oocytes. Fixed oocytes were treated with antibodies against CID (yellow), as well as the DNA dye DAPI (blue). All oocytes were from prometaphase I-enriched preparations of oocytes. (A) In a wild-type oocyte the eight CID foci are properly bioriented. (B) A *γtub37C* ^3^ */Df* mutant oocyte with bioriented kinetochores as based on CID foci. (C) A *γtub37C* ^3^ */Df* mutant oocyte with CID foci that failed to properly biorient at opposite ends of the chromosome mass. (D) A *γtub37C* ^3^ */Df* mutant oocyte with kinetochores that appear to be mono-oriented based on CID foci. (E) A *γtub37C* ^3^ */Df* mutant oocyte with clustered CID foci. (F) More than eight CID foci are observed in a *γtub37C* ^3^ */Df* mutant oocyte, suggesting loss of sister chromatid cohesion. Images are maximum intensity projections from partial Z stacks. The scale bars are in microns.

To investigate the defects in chromosome alignment in γ*tub37C* mutant oocytes, we examined the position of kinetochores using an antibody to Centromere Identifier (CID), the *Drosophila* CENP-A homolog [17]. In 93% of wild-type oocytes CID localizes to eight foci—four foci on each end of the chromosome mass oriented in opposite directions (Figure 2A, Table S1). One oocyte appeared to be in spindle assembly before kinetochores had bioriented, and one oocyte had five CID foci on a single side of the chromosome mass, most likely indicating an achiasmate chromosome was caught in the process of moving dynamically on the spindle as observed by Hughes et al. [15] (Table S1).

In γ*tub37C^3^*/*Df* mutant oocytes only 17% showed eight CID foci in the wild-type configuration (Figure 2B, Table S1). In 51% of oocytes CID foci were maloriented with more than four foci pointing towards a single direction or with foci pointing in more than two directions, indicating a frequent failure to properly biorient kinetochores during prometaphase I (Figure 2C, Table S1). We also observed CID foci that appeared to be oriented towards a single pole (Figure 2D, Table S1) or clustered together in the middle of the chromosome mass suggesting a complete failure of chromosome biorientation (Figure 2E, Table S1). Finally, in 14% of γ*tub37C^3^*/*Df* mutant oocytes more than eight CID foci were observed suggesting precocious sister chromatid separation (Figure 2F, Table S1).

Kinetochores were bioriented in only 38% of γ*tub37C^P162L^* mutant oocytes. In the majority of γ*tub37C^P162L^* mutant oocytes the CID foci were maloriented, while more than eight CID foci were detected in the remaining oocytes (Table S1). These results clearly illustrate that chromosome kinetochores fail to correctly biorient in γ*tub37C* mutant oocytes and in a few cases sister chromatid cohesion may be lost.

**Figure 3:**
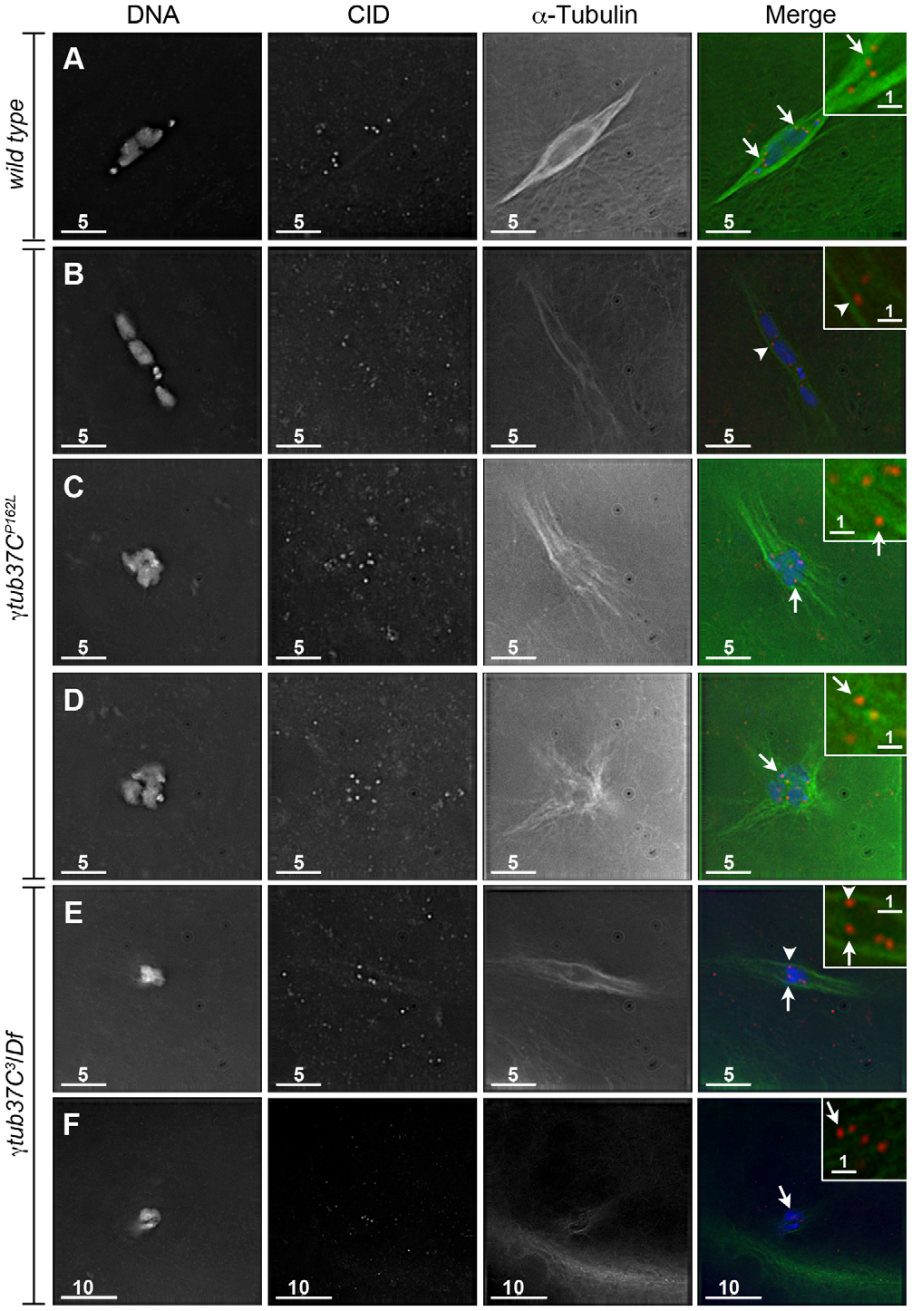
Kinetochores fail to make direct contacts to kinetochore microtubules in *γtub37C* mutant oocytes. Fixed prometaphase I oocytes were treated with antibodies against α-tubulin and CID, as well as the DNA dye DAPI. Selected regions are magnified in the insets. Insets show only CID and α-tubulin for better visualization of microtubule interactions with CID foci. (A) CID foci directly interact with kinetochore microtubules (arrows) in a wild-type oocyte. (B–D) CID foci show aberrant kinetochore microtubule attachments in *γtub37C^P162L^* mutant oocytes. Arrows point to examples of CID foci with no apparent direct microtubule interactions and the arrowhead points to an example of a CID focus with potentially a lateral microtubule interaction. (E–F) Shows spindles from *γtub37C* ^3^ */Df* mutant oocytes. The arrows point to examples of CID foci lacking clear microtubule interactions and the arrowhead points to an example of a CID focus showing only a potential lateral interaction to microtubules. Images are single Z sections to highlight the interaction of CID foci with the microtubules. Black levels were adjusted to allow better visualization of CID foci. Scale bars are in microns.

To better understand the defects in kinetochore orientation, we examined the location of CID foci in comparison to α-tubulin. In wild-type oocytes CID foci appear to interact directly with the kinetochore microtubules as shown in Figure 3A. In γ*tub37C* mutants we observed that many CID foci lacked these direct connections to microtubules, despite careful analysis of all sections of the imaged Z stacks (Figure 3B–3F). While we observed some CID foci that appeared to lack clear interaction with any microtubules (Figure 3C–3F), it appeared that other CID foci may potentially have lateral interactions with microtubules (Figure 3B, 3E).

These data suggest that γTub37C plays an important role in allowing microtubules to attach properly to kinetochores. The disruption of microtubule attachments to some kinetochores in γ*tub37C* mutants may result in kinetochores failing to undergo proper biorientation [18], as well as contributing to the observed spindle and chromosome alignment defects.

### Live imaging reveals dynamic changes in spindle and chromosome configurations in γ*tub37C^P162L^* mutant oocytes

To better understand the defects observed in the γ*tub37C^P162L^* mutant oocytes, we examined chromosome movement and spindle dynamics in live oocytes as described in Hughes et al. [15]. Video S1 shows a prometaphase I spindle from a wild-type oocyte. The chromosomes are aligned on the spindle midzone and the spindle is bipolar, consistent with fixed images and previous live-imaging studies [15]. The spindle and chromosomes remain stable for over an hour of observation.

We were able to image nuclear envelope breakdown and successful formation of a spindle-like structure in five γ*tub37C^P162L^* mutant oocytes. In all five the resulting spindles were either barrel-like or failed to maintain a tapered bipolar shape throughout the period of imaging (Video S2). The ends of the spindles were frequently observed to wave back and forth, and the entire spindle often moved in different directions. The chromosomes also moved quickly into different configurations, at times showing no alignment on the spindle midzone.

**Figure 4:**
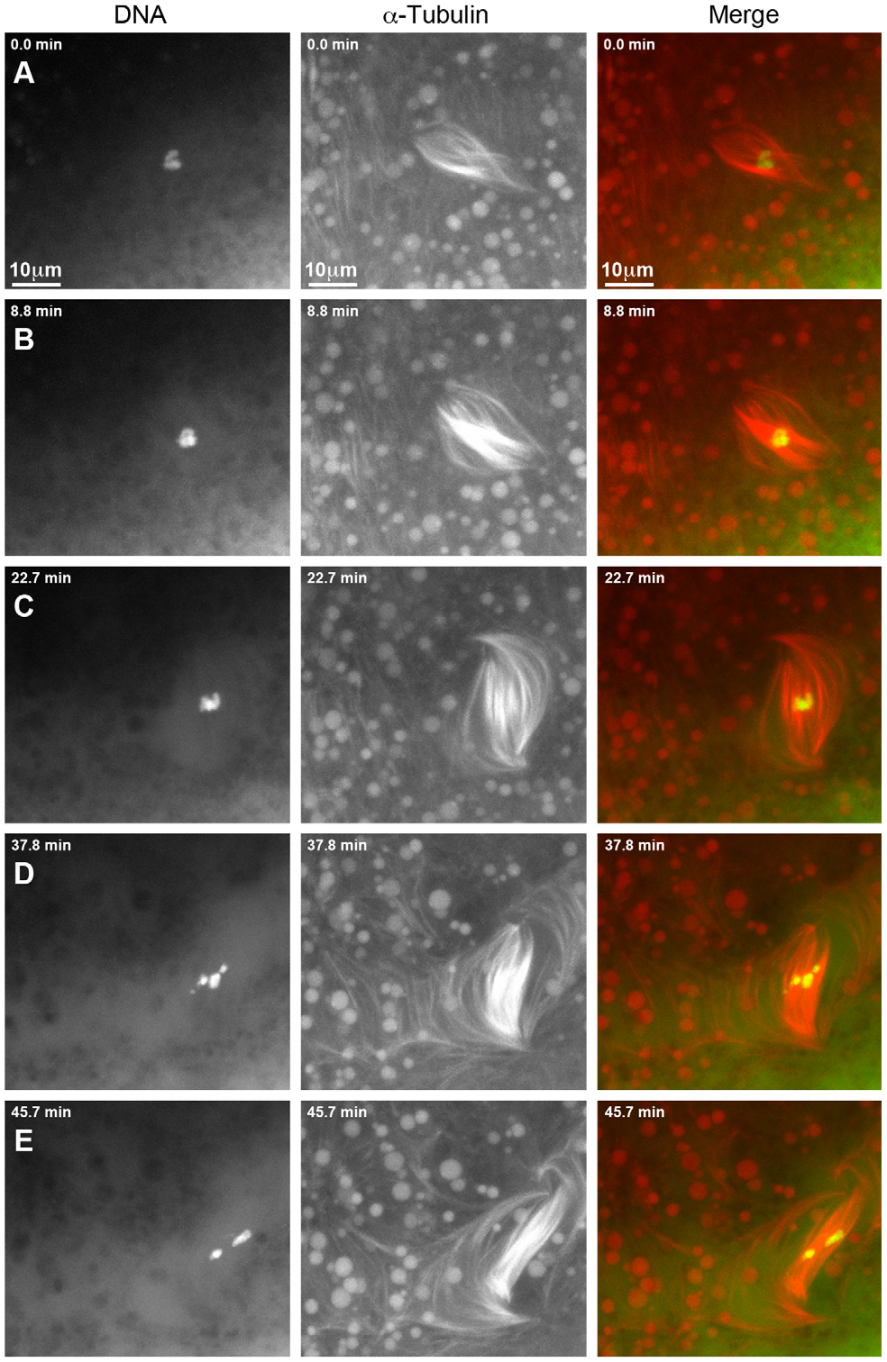
Defects in spindle morphology and chromosome positioning are observed in a living *γtub37C^P162L^* mutant oocyte. Shown is a time-lapse of a spindle from a living prometaphase I *γtub37C^P162L^* mutant oocyte. OliGreen labels the DNA (green) and rhodamine-conjugated-tubulin labels the microtubules (red). The time-lapse is shown in Video S3. (A) At the start of the time-lapse the chromosomes are associated, but the spindle is wide and lacks tapered poles. (B) At 8.8 minutes the spindle has changed shape due to the movement of the microtubules. (C) At 22.7 minutes the microtubules continue to move and the whole spindle has rotated clockwise. The chromosomes also appear to have changed position. (D) At 37.8 minutes the chromosomes have moved apart, the spindle further broadens, and microtubules are shed into the cytoplasm. (E) By the end of Video S3 at 45.7 minutes the chromosomes have formed two masses. The spindle has narrowed and rotated an additional 30° clockwise. Images are maximum intensity projections from Z stack and scale bars are in microns.

For an additional 16 γ*tub37C^P162L^* mutant oocytes the prometaphase I spindle was already present at the start of live-imaging. These spindles displayed many of the same phenotypes that were observed for the spindles that formed after observation of nuclear envelope breakdown. Video S3 shows one such prometaphase I spindle and Figure 4 shows selected still images from this video. At the beginning of Video S3 the DNA is associated with the spindle midzone but the spindle is abnormally wide and barrel-like (Figure 4A). In Figure 4B and 4C the chromosomes stay associated but change in shape, suggesting movement of the chromosomes with respect to one another. The spindle rotates almost 60° clockwise and individual microtubules move rapidly. By 37.8 minutes after imaging began the microtubules appear to be shed into the cytoplasm and the bivalents begin to separate on the spindle (Figure 4D). At the end of imaging (45.7 minutes), the chromosomes have split into two distinct masses (Figure 4E). The spindle has turned another 30° clockwise and starts to drift out of the field of view. The dynamic movement of individual microtubules and the shedding of multiple microtubules into the cytoplasm is not commonly observed in wild-type oocytes after the completion of nuclear envelope breakdown [15]. These microtubule movements imply that individual microtubule bundles are not maintained in an organized bipolar spindle in γ*tub37C^P162L^* mutant oocytes, suggesting that γTub37C is required for stabilizing microtubules relative to one another within the spindle.

Spindles in wild-type oocytes typically remain fairly stationary for long periods of imaging. However, spindles in γ*tub37C^P162L^* mutant oocytes frequently shifted position and orientation quickly which hampered imaging for long time periods. As shown in Video S4, movement of the ends of the thin and long barrel-like spindle eventually leads to the spindle moving toward the top edge of the focal area. After re-centering the spindle within the focal plane, the spindle and the chromosomes within the spindle continue to move rapidly. The spindle becomes progressively thinner during these movements until it eventually dissolves and the moving chromosomes disperse. These changes in spindle position may be due to the rapid movement of the spindle microtubules or the possible role of γTub37C in maintaining the cortical microtubules that anchor the meiosis I spindle.

When examining single time points from live-imaging of *γtub37C* mutant oocytes most, if not all, of the spindle and chromosome configurations observed in fixed images could be identified. The reason we observe both normal and aberrant spindles in fixed images is that they represent transient intermediates in dynamically changing but structurally flawed spindles. For example, Video S2 shows a spindle that gains and loses tapered bipolarity during the course of imaging. Shortly after nuclear envelope breakdown a tapered bipolar spindle is formed. Around 40.3 minutes the chromosomes begin to spread out across the spindle midzone, followed by the spindle forming into a wide barrel. Despite continued reshuffling of the chromosomes across the spindle midzone the spindle regains tapered bipolarity by 50.9 minutes. The bottom pole of the spindle then widens and refocuses a second time before a bipolar spindle reforms and is maintained for the remainder of imaging.

Due to problems inherent in finding the meiotic spindle, imaging of γ*tub37C^3^/Df* mutant oocytes proved very difficult. We were unable to acquire any quality recordings of γ*tub37C^3^/Df* mutant oocytes despite numerous efforts.

The results from live-imaging suggest that γTub37C is involved in bipolar spindle assembly and maintenance as well as chromosome and spindle positioning during prometaphase I. Additionally, live-imaging demonstrates that mutants can both lose and regain wild-type spindle and chromosome morphology. This suggests that the array of phenotypes observed in fixed preparations are not due to the spindle progressively deteriorating during the progression to metaphase I arrest but rather represents different stages of the dynamic spindle recovery and dissolution process.

### Many γ*tub37C* mutant oocytes can recover spindle bipolarity and the chromosomes can properly congress by metaphase I arrest

**Figure 5:**
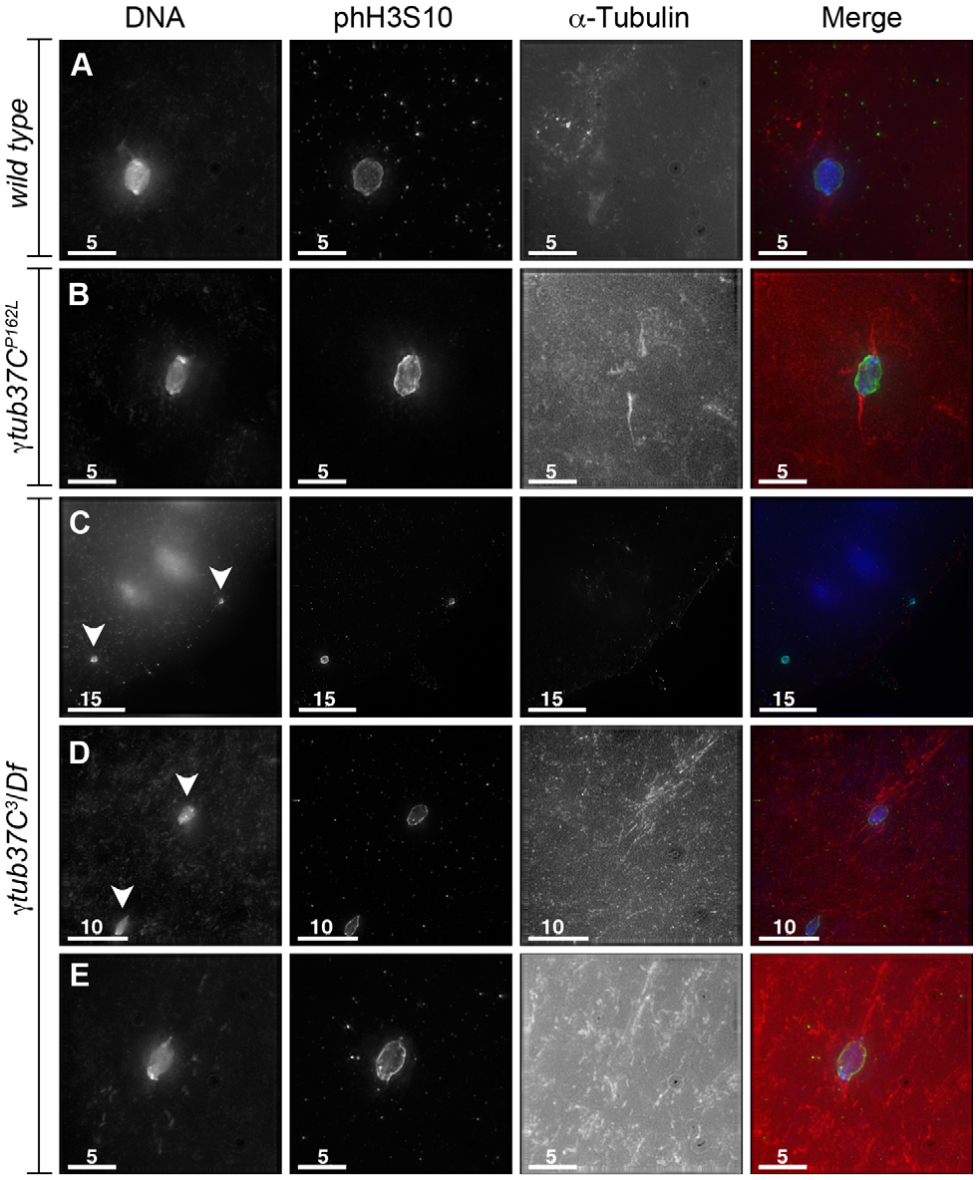
Oocytes with a missense mutation in *γtub37C* mostly recover by metaphase I arrest while a null mutation causes splitting of the chromosomes. Fixed oocytes were treated with antibodies against α-tubulin and histone H3 phosphorylated at serine 10 (phH3S10), as well as the DNA dye DAPI. Oocytes were from virgin mothers (to enrich for metaphase I arrested oocytes). (A) A wild-type metaphase I-arrested oocyte with chromosomes that have congressed to the metaphase plate to form a lemon-shaped morphology. The bipolar spindle has shortened. (B) An oocyte from a *γtub37C^P162L^* mutant oocyte displays properly congressed chromosomes, but the spindle is extremely narrow. (C) A *γtub37C^3^/Df* mutant oocyte with a DNA mass that has split into 2 parts (arrowheads) which have moved far apart in the cytoplasm. Microtubules do not appear to be associated with the chromosome masses. (D) A *γtub37C^3^/Df* mutant oocyte with a chromosome mass split into two parts (arrowheads) that are relatively close together. Each piece is associated with its own set of microtubules. (E) A *γtub37C^3^/Df* mutant oocyte that has achieved a wild-type metaphase I chromosome morphology and a bipolar spindle. All images are maximum intensity projections from Z stacks. Scale bars are in microns.

Fixed preparations of virgin females four to five days post-eclosion are highly enriched for oocytes arrested at metaphase I (Table 3) [14]. In wild-type oocytes the achiasmate chromosomes congress back to the metaphase plate and the chromosomes form a lemon-shaped configuration for metaphase I arrest at stage 14 (Figure 5A) [14]. Chromosomes that had congressed into a lemon-like shape on the metaphase plate were observed in 96% of wild-type oocytes (Table 3).

**Table 3:** Metaphase I preparations: DNA configurations.

Genotype	Prometaphase aligned^1^	Metaphase-like^2^	Autosomal slippage^3^	Abnormal chromosome mass^4^	Divided chromosome mass^5^	Abnormal threads^6^	Total
*+/+*	2	45	0	0	0	1	47
*γtub37C^P162L^*	2	24	3	7	3	6	39
*γtub37C* *^3^* */Df*	2	23	0	4	37	4	66

1 Chromosomes are symmetrically arranged on the meiotic spindle, see Figure 1A.

2 Chromosomes have all congressed back to the metaphase plate to form a lemon-shaped configuration, see Figure 5A.

3 Chiasmate chromosomes are randomly arranged on the spindle, see Figure 1B.

4 All the chromosomes are associated but the chromosomal mass is abnormal in structure, see Figure 1E.

5 Chromosomes are not present on the same spindle-like structure, see Figure 5C.

6 Threads staining with DAPI and an antibody to histone H3 phosphorylated at serine 10 are present that do not connect chromosomes.

Despite the abnormal chromosome morphologies observed during prometaphase I, the chromosomes in 62% of γ*tub37C^P162L^* mutant oocytes successfully congressed to the metaphase plate to form lemon-like DNA structures (Figure 5B, Table 3). However, 8% of γ*tub37C^P162L^* mutant oocytes displayed chromosome masses that had split into 2 or more parts (discussed below). In 18% of γ*tub37C^P162L^* mutant oocytes chromosomes were associated but formed irregular, non-lemon-shaped configurations (Table 3).

Chromosome morphology at metaphase I arrest was also examined in oocytes from virgin *γtub37C^3^/Df* mutant females. Metaphase I DNA configurations were observed in 35% of *γtub37C^3^/Df* mutant oocytes, demonstrating that chromosomes can successfully congress to the metaphase plate in some oocytes lacking γTub37C (Figure 5E, Table 3). For *γtub37C^3^/Df* mutant oocytes, we noted a propensity for the chromosome mass to split into multiple pieces. This phenotype was observed for 56% of oocytes (Figure 5C–5D, Table 3). In some oocytes the chromosome masses were near each other (Figure 5D) but in many others the chromosome masses were dispersed throughout the cytoplasm (Figure 5C). The phH3S10 antibody facilitated the identification of all the chromosomes, as some chromosomes were not associated with clear spindles (See Figure 5C for an example).

**Figure 6:**
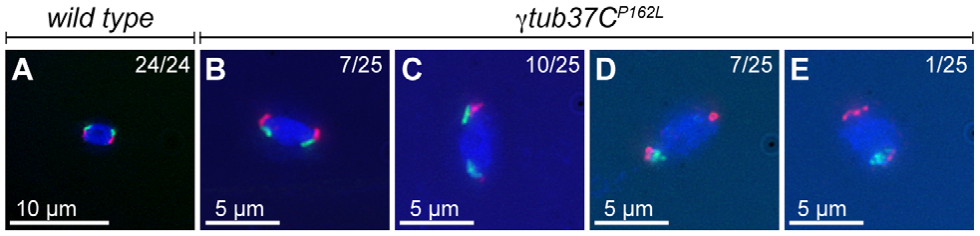
At metaphase I chromosomes congress randomly in *γtub37C^P162L^* mutant oocytes. Shown is localization of FISH probes to the 359 base pair (bp) heterochromatic repeat on the *X* (green) and to the AATAT heterochromatic repeats on a large region of the *4^th^* chromosomes and a small region of the *X* chromosomes (red), as well as DAPI (blue) in oocytes from metaphase I-enriched preparations. (A) A wild-type oocyte with an *X* and *4^th^* chromosome on each end of the chromosome mass indicating proper alignment of chromosomes in preparation for segregation at anaphase I. (B) A *γtub37C^P162L^* mutant oocyte with *X* and *4^th^* chromosomes properly positioned on each end of the chromosome mass. (C) A *γtub37C^P162L^* mutant oocyte with both *4th* chromosomes on the same side of the chromosome mass. (D) A *γtub37C^P162L^* mutant oocyte with both *X* chromosomes on the same side of the chromosome mass. (E) A *γtub37C^P162L^* mutant oocyte with the *4th* and *X* chromosomes on opposite sides of the chromosome mass. Numbers indicate the number of oocytes displaying each chromosome configuration over the total oocytes scored. For the probe recognizing the AATAT heterochromatic repeat, assignment of signal to the *4^th^* or *X* chromosomes was based on the size of the signal and proximity to the 359 bp probe signal. Images are projections from Z stacks and scale bars are in microns.

Although chromosomes successfully congressed to the metaphase plate in most γ*tub37C^P162L^* mutant oocytes, they failed to properly orient in preparation for segregation at anaphase I. Using Fluorescent In-Situ Hybridization (FISH) probes that recognize the *X* and *4^th^* chromosomal heterochromatin, we observed in wild-type oocytes that the *X* and *4^th^* chromosomes were properly positioned on each half of the chromosome mass in 100% of oocytes (Figure 6A), which is consistent with previous studies [14]. This configuration was observed in only 28% of γ*tub37C^P162L^* mutant oocytes (Figure 6B). In 40% of γ*tub37C^P162L^* mutant oocytes the *4^th^* chromosomes were associated on the same side of the chromosome mass while the *X*s were properly segregated (Figure 6C). In 28% of oocytes the *X* chromosomes were associated on the same side of the chromosome mass while the *4^th^* chromosomes were segregated correctly (Figure 6D). Finally, in 4% of γ*tub37C^P162L^* mutant oocytes the *X* and *4^th^* chromosomes were on opposite ends of the chromosome mass (Figure 6E). These results show that while γTub37C is not involved in chromosome congression, it does play a role in ensuring that the chromosomes are packaged correctly when they do congress. The aberrant threads observed during prometaphase I were mostly absent during metaphase I in *γtub37C* mutant oocytes (15% for *γtub37C^P162L^* and 6% for γ*tub37C^3^/Df*, Table 3). Whether the aberrant threads were resolved or simply packaged into the chromosome mass during congression is unknown.

In wild-type oocytes the spindle becomes shorter as the chromosomes congress, which results in a small, bipolar, tapered spindle, similar to the spindle in mouse oocytes [14], [19]. Bipolar spindles were observed in 87% of wild-type oocytes from four to five day-old virgin wild-type females (Table 4). While the γ*tub37C^P162L^* mutation caused spindle abnormalities during prometaphase I in 70% of oocytes (Table 2), many mutant oocytes were able to recover to form spindles resembling wild-type metaphase I-arrested spindles. Short, tapered, bipolar spindles were observed in 54% of γ*tub37C^P162L^* and 8% of *γtub37C^3^/Df* mutant oocytes (Table 4). In 35% of *γtub37C^3^/Df* oocytes the spindle was reduced to a large microtubule bundle projecting from each end of the chromosome mass (Table 4). This spindle phenotype was also observed in 21% of γ*tub37C^P162L^* mutant oocytes (Figure 5B, Table 4). In 33% of *γtub37C^3^/Df* oocytes and 5% of γ*tub37C^P162L^* mutant oocytes, no apparent microtubules were associated with the chromosomes. This phenotype was often associated with chromosome masses that had split apart. The remaining γ*tub37C^P162L^* and *γtub37C^3^/Df* mutant oocytes displayed spindles that were abnormal in ways similar to those from prometaphase I-enriched preparations, such as barrel-like spindles, monopolar spindles, and microtubule aggregations lacking directionality (Table 4).

**Table 4:** Metaphase I preparations: spindle configurations.

Genotype	Bipolar	Monopolar^1^	Barrel-like^2^	Narrow spindle^3^	Microtubules bundles with no directionality^4^	None^5^	Total
*+/+*	41	6	0	0	0	0	47
*γtub37C^P162L^*	21	6	1	8	1	2	39
*γtub37C* *^3^* */Df*	5	7	4	23	5	22	66

1 One pole is focused while the other end of the spindle is unfocused, see Figure 1B.

2 Microtubules are aligned in the same direction but both poles are unfocused, see Figure 1C.

3 Only isolated thick bundles of microtubules appear to be projecting from the chromosome mass, see Figure 5B.

4 Microtubules are not aligned in a single direction, see Figure 1E.

5 Clear microtubules were not associated with the chromosomes despite good antibody penetration.

These results suggest that γTub37C plays a less pivotal role in spindle maintenance and chromosome positioning during metaphase I. The ability of chromosomes to congress to the metaphase plate in many *γtub37C* mutant oocytes suggests that chromosome congression during metaphase I is mediated by a mechanism that is different from chromosome positioning during prometaphase I and that does not require a robust prometaphase I spindle.

### D-TACC is mislocalized on γ*tub37C^P162L^* mutant spindles during prometaphase I and metaphase I

**Figure 7:**
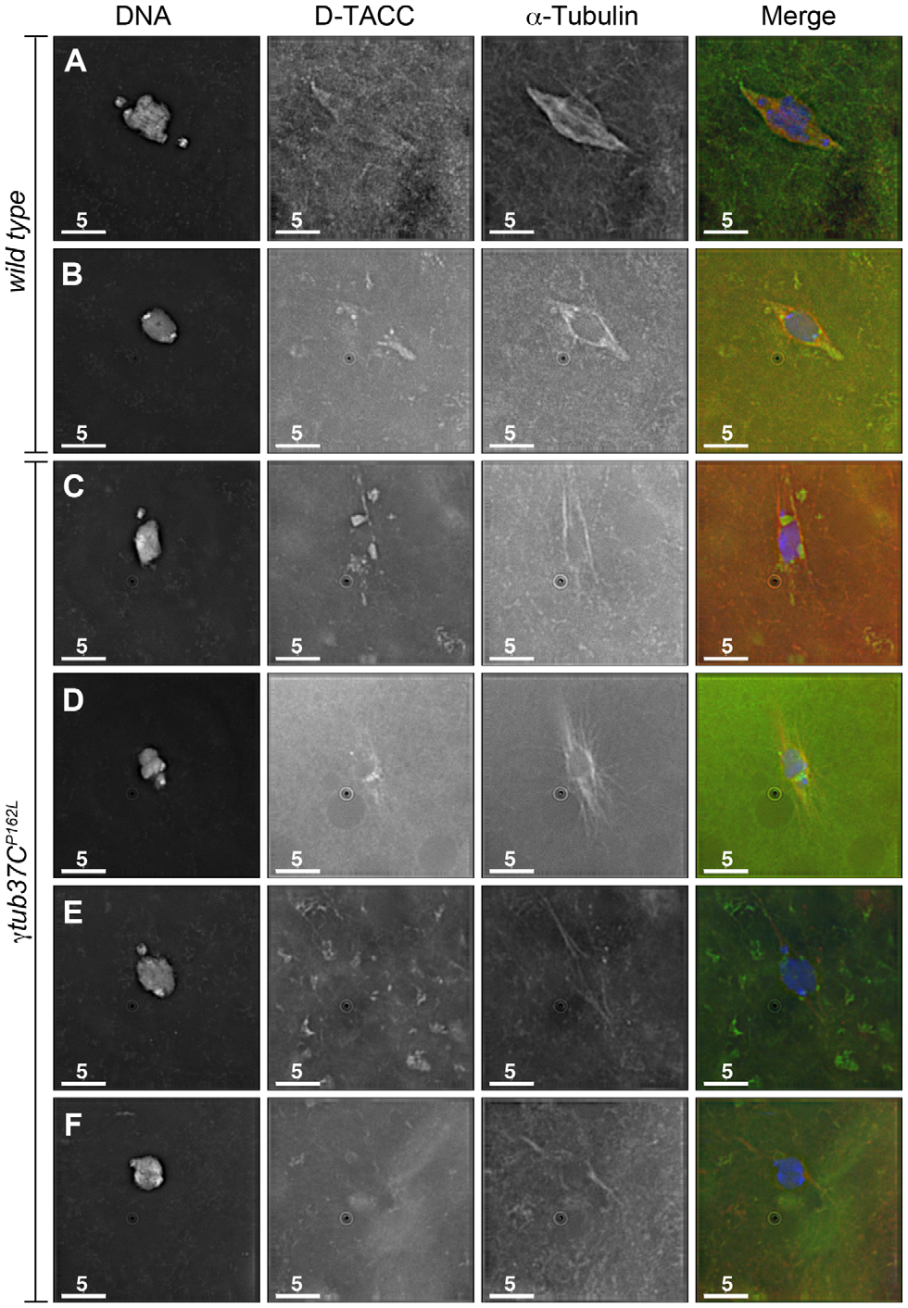
The spindle pole component D-TACC is mislocalized in *γtub37C^P162L^* mutant oocytes. Oocytes were immuno-stained to D-TACC (green), α-tubulin (red) and DAPI (blue). (A) A wild-type prometaphase I spindle with polar and diffuse D-TACC localization. (B) A wild-type metaphase I spindle with D-TACC staining at the spindle poles and diffuse staining along the microtubules. (C) Large, bright patches of D-TACC cover part of the spindle while D-TACC is absent in other regions in a prometaphase I *γtub37C^P162L^* mutant oocyte. (D) Small puncta of D-TACC are present near the DNA, but D-TACC is extremely weak or absent near the poles in a prometaphase I *γtub37C^P162L^* mutant oocyte. (E) D-TACC is absent or extremely weak from the spindle in a prometaphase I *γtub37C^P162L^* mutant oocyte despite strong localization of D-TACC to cytoplasmic structures. (F) A metaphase I spindle from a *γtub37C^P162L^* mutant oocyte lacking clear D-TACC staining. Shown are single Z slices since projection of the entire Z stack results in images with high background in the D-TACC channel. For the examples of *γtub37C^P162L^* mutant oocytes, spindles that displayed directionality were analyzed to rule out D-TACC mislocalization being caused by lack of a clear spindle. Scale bars are in microns.

In *Drosophila* oocytes the spindle pole protein D-TACC is required for maintaining the bipolarity of the meiosis I spindle [20]. Spindles fail to maintain bipolarity in *d-tacc* mutant oocytes [20]. In *Caenorabditis elegans* embryos, γ-tubulin plays a role in localizing TACC (called TAC-1 in *C. elegans*) to the spindle poles [21]. *Drosophila* oocytes with a mutation in the Cdc2 subunit, Cks/Suc1, display defects in chromosome alignment and spindle morphology as well as mislocalization of D-TACC [22]. These results suggest that D-TACC mislocalization can also cause spindle defects. We examined whether D-TACC localization was disrupted in γ*tub37C^P162L^* mutant oocytes. Using an antibody to D-TACC, Cullen and Ohkura [20] reported that D-TACC primarily localizes to the poles of the meiosis I spindle with diffuse staining of spindle microtubules in a few oocytes. In contrast, we observed this additional diffuse localization in the majority of wild-type prometaphase I and metaphase I spindles we examined (Figure 7A and 7B). Moderate to strong diffuse spindle and polar staining was observed in 23/36 (64%) prometaphase I and 34/45 (76%) metaphase I wild-type oocytes. Weak diffuse spindle and polar staining was observed in an additional 7 (19%) and 4 (9%) prometaphase I and metaphase I spindles, respectively. In the remaining 6 (17%) prometaphase I and 5 (11%) metaphase I wild-type oocytes D-TACC could either not be detected on the spindle or the background staining was too high to assign a definitive localization pattern. One metaphase I oocyte showed staining primarily to the poles and one showed patchy D-TACC staining (described below).

In γ*tub37C^P162L^* mutant oocytes, D-TACC localization was frequently abnormal. In 38/63 (60%) prometaphase I oocytes, the D-TACC localization was patchy with large, bright foci in some regions and no staining in other regions of the spindle (Figure 7C). In 8/63 (13%) prometaphase I γ*tub37C^P162L^* mutant oocytes, small, punctate spots of staining were observed along only part of the spindle, often close to the chromosomes (Figure 7D). In 12/63 (19%) of prometaphase I γ*tub37C^P162L^* mutant oocytes, D-TACC was absent or highly reduced on the spindle (Figure 7E). Only 5/63 (8%) prometaphase I oocytes displayed the diffuse microtubule and polar D-TACC localization observed in wild-type oocytes. For the 49 γ*tub37C^P162L^* mutant oocytes examined from metaphase I-enriched preparations, 12 (24%) displayed patchy D-TACC localization, 6 (12%) displayed punctuate localization, 29 (59%) showed reduced or absent staining (Figure 7F), and only 2 (4%) displayed D-TACC localization similar to wild-type. Since D-TACC is required for the proper formation of spindle poles, mislocalization of this spindle assembly factor could contribute to the lack of defined spindle poles in *γtub37C* mutant oocytes.

### Mutations in γ*tub37C* cause defects in nuclear positioning

During our attempts to examine living *γtub37C* mutant oocytes, we noticed that the oocyte nucleus was mislocalized within the cytoplasm with respect to the dorsal appendages. The mislocalization of the oocyte nucleus was not surprising since cortical microtubules are required for nuclear positioning [23]. Additionally, mutations in γ-tubulin ring components (γTURCs) have been reported to affect bicoid RNA localization to the anterior cortex of *Drosophila* oocytes, which is a process dependent on microtubules [24].

In DAPI-only fixed preparations, 98% of wild-type oocyte nuclei were localized to the anterior third of the oocyte near the nurse cells during stages 11 and 12 (Table S2). In contrast, oocyte nuclei were mislocalized in 22% of γ*tub37C^P162L^* mutant oocytes during stages 11 and 12 (Table S2). In *γtub37C^3^*/*Df* mutant oocytes, only 65% of oocyte nuclei were in the anterior third of the oocyte while 26% were located in the middle of the oocyte and 9% of oocyte nuclei were located in the posterior third of the oocyte (Table S2).

In wild-type stage 13 and 14 oocytes, 100% of the oocyte nuclei were located near the dorsal appendages in the anterior one-third of the oocyte (Table S2). Meanwhile, in γ*tub37C^P162L^* mutant oocytes, 60% of the oocyte nuclei were located in the anterior portion of the oocyte and 40% were located in the middle or posterior of the oocyte (Table S2). In γ*tub37C^3^/Df* mutant oocytes, only 37% of the oocyte nuclei were in the anterior third of the oocyte at stages 13 and 14 (Table S2). In 43% of γ*tub37C^3^/Df* mutant oocytes, the nucleus was in the middle third of the oocyte and in 20% the oocyte nucleus was in the posterior portion of the oocyte (Table S2). Our data suggest that γTub37C plays a role in nuclear positioning, most likely by affecting the cortical microtubules required to stabilize the oocyte nuclei in the anterior part of the oocyte during prophase I and after spindle formation.

### Endogenous γTub37C localizes to the meiosis I spindle

As noted above, multiple laboratories have failed to detect endogenous γTub37C on the meiosis I spindle [6], [8], [11], [25]. More recently, Endow and Hallen [10] reported that an overexpressed transgenic GFP-tagged γTub37C protein localized to a subset of meiosis I spindles. This experiment had the caveat that the γTub37C-GFP construct was driven not by the genomic *γtub37C* promoter but rather by the *ncd* promoter, and endogenous γTub37C was still present in these oocytes. Thus, the γTub37C-GFP construct was likely not present at endogenous levels, raising the possibility that the localization of γTub37C-GFP does not represent the localization of endogenous γTub37C. In order to address these issues and demonstrate the presence of endogenous γTub37C on the meiosis I spindle, we re-examined γTub37C localization using multi-spectral imaging.

We examined the localization of endogenous *γ*Tub37C using an antibody raised against the C-terminal 17 amino acids of *γ*Tub37C (DrosC) [26]. Processing and acquisition of the images was optimized for the highest possible detection efficiency of *γ*Tub37C staining and the maximum possible rejection of signal from other fluorescent labels and autofluorescence. The method of choice for these applications is multispectral imaging [27], [28], [29]. This method provides both high signal-to-noise imaging of multiple fluorophores, as well as residuals-based verification of fitting quality. This allows for detection and removal of even small amounts of bleedthrough or autofluorescence in confocal images. In this case, it permits accurate determination of the presence or absence of γTub37C in the company of α-tubulin, which is in much higher abundance.

**Figure 8:**
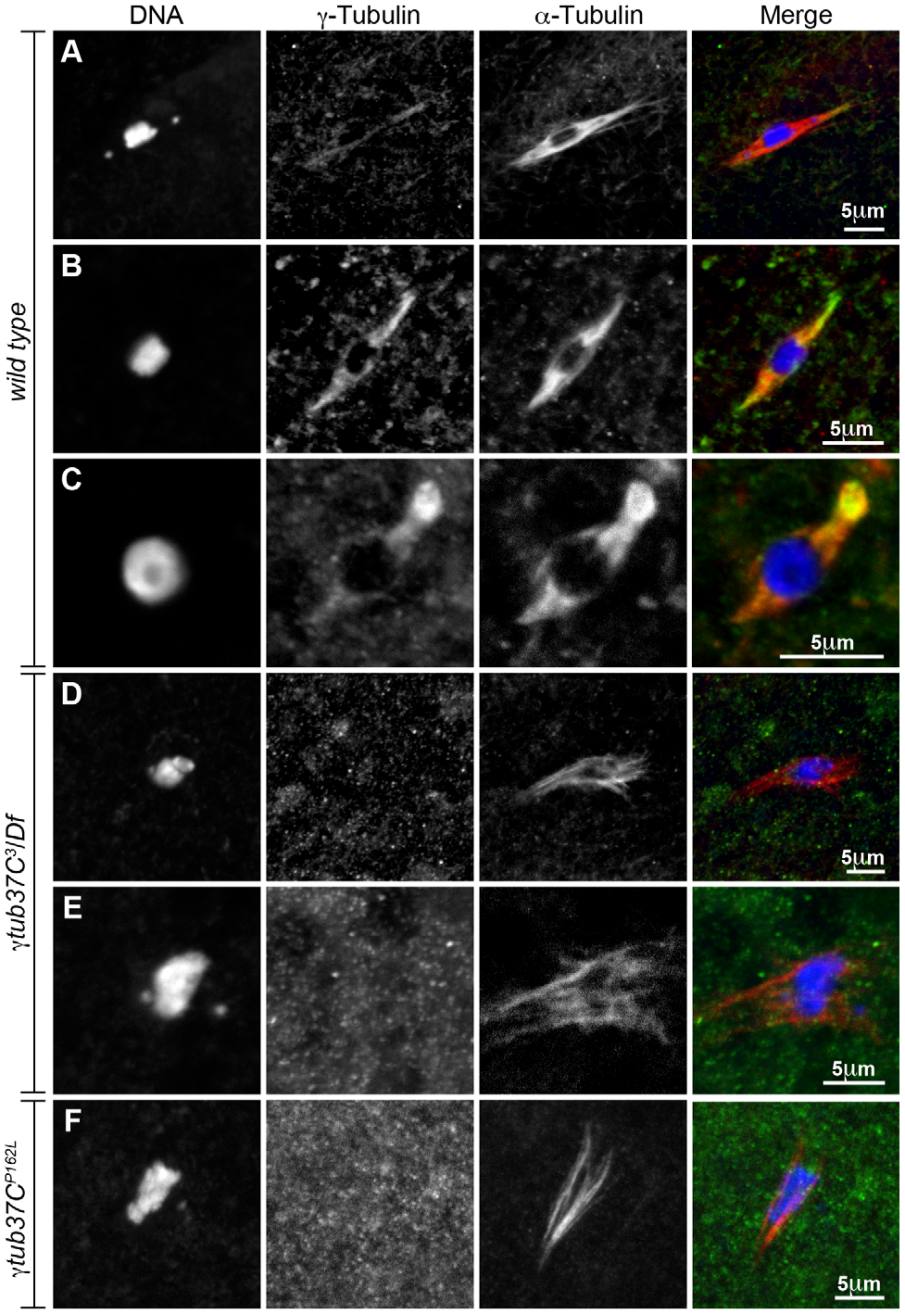
Endogenous γTub37C is present on the meiosis I spindle in ***Drosophila*** **oocytes.** The DrosC γTub37C-specific and α-tubulin antibodies in A–F were acquired simultaneously using 488 excitation, while Hoescht (A–E) or DAPI (F) was acquired separately using the exact same Z-stack parameters. (A and B) γTub37C localizes to wild-type prometaphase I spindles in a pattern similar to α-tubulin (N = 20). (C) γTub37C continues to localize to the spindle microtubules in a wild-type metaphase I oocyte (N = 10). (D–E) The γTub37C-specific antibody fails to recognize the spindle in *γtub37C^3^*/*Df* mutant oocytes (N = 18). (F) γTub37C is not detected on a spindle from a *γtub37C^P162L^* mutant oocyte (N = 20). Images are projections of a few Z slices. Scale bars are in microns.

We observed *γ*Tub37C antibody localizing to all the wild-type meiosis I spindles examined while Endow and Hallen [10] failed to detect γTub37C-GFP on the meiosis I spindle in some oocytes (Figure 8A–8C). Rather than staining predominantly at spindle poles with weaker staining on the remaining spindle as is observed in embryos [6], [30], a uniform signal was observed over the entire spindle for both prometaphase I (Figure 8A–8B) and metaphase I spindles (Figure 8C). This staining is similar to γ–tubulin staining in mitotic and meiotic cells in other systems [31]. Indeed, *γ*Tub37C co-localized with the α-tubulin antibody signal, although *γ*Tub37C localization was sometimes seen to extend slightly past the α-tubulin signal to form more defined spindle poles (Figure 8B).

To ensure that the DrosC anti-γTub37C antibody is specific for γTub37C we examined γ*tub37C^3^*/*Df* mutant oocytes. We focused our analysis on those oocytes that formed a chromatin-associated microtubule structure to rule out the possibility of failing to detect γTub37C simply due to lack of a “spindle.” All γ*tub37C^3^*/*Df* mutant oocytes examined failed to show detectable DrosC antibody staining on the spindle (Figure 8D–8E). This result suggests that the DrosC antibody is specific for the C-terminus of γTub37C since the γ*tub37C^3^* allele results in a 160 amino-acid C-terminal truncation of the γTub37C protein. These data clearly demonstrate, contrary to previous studies using other γTub37C antibodies, that endogenous γTub37C is present on the meiosis I spindle of *Drosophila* oocytes.

We also examined the localization of γTub37C in γ*tub37C^P162L^* mutant oocytes and failed to detect γTub37C on the meiosis I spindle in all γ*tub37C^P162L^* mutant oocytes examined (Figure 8F). Based on results from Western blot analysis, γTub37C does appear to be expressed in γ*tub37C^P162L^* mutant ovaries (Figure S3). While the γ*tub37C^P162L^* mutation appears to be a strong loss-of-function allele, it causes phenotypes that are slightly weaker at prometaphase I and noticeably weaker in terms of the split chromosome mass phenotype at metaphase I compared to the null allele (γ*tub37C^3^*). For these reasons, we expected to see only a reduced level of γTub37C on the meiotic spindle. A small amount of γTub37C may be present on the meiosis I spindle in γ*tub37C^P162L^* mutant oocytes, but it may be below our level of detection. Another possibility is that γTub37C has a function not directly associated with the spindle microtubules to regulate meiosis I, and the γ*tub37C^P162L^* mutation does not completely abrogate this function.

## Discussion

Our analysis both confirms the conclusion of Tavosanis et al. [8] that γTub37C is required for the organization of the female meiotic spindle and extends that conclusion in several very important ways. First, *γtub37C* mutant oocytes show strong defects in kinetochore biorientation during prometaphase I. Indeed, in *γtub37C* mutant oocytes many kinetochores appear to lack typical kinetochore microtubule attachments, suggesting that γTub37C plays an essential role in initiating or maintaining the kinetochore microtubule attachments that are required for properly positioning the chromosomes on the meiotic spindle. Our demonstration of a role for γTub37C in mediating kinetochore microtubule interactions in meiosis is consistent with the observation by others that in γ-tubulin depleted S2 cells subjected to cold-induced microtubule depolymerization, kinetochore-driven microtubule regrowth is delayed [32]. Additionally, in HeLa cells the γ-tubulin Ring Complex is recruited to unattached kinetochores and is required for nucleation of kinetochore microtubules [33]. These studies suggest that γ-tubulin may play a role in kinetochore microtubule nucleation during acentriolar meiosis similar to that in mitosis.

Second, although the defects in spindle assembly exhibited by *γtub37C* mutants are first observed soon after nuclear envelope breakdown and remain severe throughout prometaphase I, normal spindle morphology and chromosome alignment can be lost and regained throughout prometaphase I. Indeed, spindle microtubules in living *γtub37C^P162L^* mutant oocytes often appeared to be rapidly moving and microtubule bundles were frequently seen being shed into the cytoplasm. These observations suggest that γTub37C plays an important role in stabilizing existing microtubules within the spindle. The visualization of spindles splitting apart and the subsequent dissociation of the chromosomes from the spindle during prometaphase I by live imaging suggests a mechanism for the split chromosome mass phenotype that is often observed in fixed images.

Third, and quite surprisingly, many metaphase I arrested oocytes appear to partially or even fully recover normal spindle and chromosome morphologies, but the chromosomes fail to orient correctly in the metaphase I chromosome mass. The fact that the highly morphologically abnormal prometaphase I spindles in *γtub37C* mutant oocytes can nonetheless progress to form metaphase I spindles that are relatively normal in appearance may reflect the existence of redundant mechanisms that can facilitate spindle assembly. The existence of such redundant mechanisms of spindle assembly is supported by the observation that when *γ*-tubulin is decreased in *Drosophila* mitotic cells, spindle assembly is delayed but a mitotic spindle does eventually form [34], [35].

Fourth, we also uncovered a previously unidentified role for γTub37C in nuclear positioning. The spindles in living *γtub37C^P162L^* mutant oocytes rapidly changed position and orientation within the cytoplasm. Additionally, fixed preparations showed that the oocyte nucleus was sometimes mislocalized to the middle and posterior portion of the oocyte. Cortical microtubules likely play a role in maintaining spindle position within the cytoplasm and a role for *γ*Tub37C in regulating these microtubules seems probable [23]. A similar defect in spindle positioning was observed in live oocytes carrying a mutation in *ncd*, a gene encoding a kinesin motor protein required for bundling the microtubules of the meiotic spindle [11], [23].

Finally, we observed two previously undescribed defects in chromatin morphology in *γtub37C* mutant oocytes at prometaphase I. Chromosomes at this stage were often obviously morphologically abnormal and thread-like chromatin projections emanating along microtubules were frequently observed. The observed disruption in chromatin morphology, as well as the potential disruption of the chromatin threads that normally connect achiasmate chromosomes [15], may well underlie the failure of chromosomes to properly package into the chromosome mass at metaphase I in *γtub37C^P162L^* mutant oocytes. Whether the unusual threads and chromosome morphology defects are an indirect effect of the abnormal spindles or whether *γ*Tub37C plays a more direct role in mediating chromosome morphology remains to be elucidated. Abnormal movement of the chromosomes due to a lack of kinetochore microtubules could potentially result in homologs moving so far apart that the chromatin threads sever or that the threads from different chromosomes become entangled and pulled out when chromosomes move around aberrantly.

While consistent with the observations of Tavosanis et al. [8] and Jang et al. [36], our conclusion that γTub37C is required for the assembly and function of the meiosis I spindle conflicts with those of Wilson and Borisy [9] and Endow and Hallen [10]. Both groups concluded that γTub37C did not play an essential role in spindle assembly and maintenance during meiosis I. We argue that there are two major causes for this discrepancy. First, Wilson and Borisy [9] and Endow and Hallen [10] based their conclusions on the observation of apparently normal spindles in some mutant oocytes. The formation of even a few bipolar spindles in *γtub37C* mutant oocytes, even for those homozygous for the weak allele of *γtub37C* used by Endow and Hallen [10], led these authors to conclude that γTub37C must not be essential for spindle formation. However, we have shown above that such apparently normal spindles are transient intermediates in a process of highly dysfunctional spindle assembly. Second, while we examined prometaphase I and metaphase I oocytes separately, Wilson and Borisy [9] mainly observed newly laid eggs or activated oocytes, often from virgin females. These preparations would be highly enriched in metaphase I-arrested oocytes and later stages of meiosis. Since many of the spindle defects observed in prometaphase I are partially or fully rectified by metaphase I, the failure to observe spindle defects in a population of oocytes enriched for metaphase I figures is not surprising. Moreover, although metaphase I appeared relatively wild-type in *γtub37C* mutant oocytes based on DAPI and α-tubulin staining, FISH revealed that chromosome alignment was still defective within the chromosome mass.

**Figure 9:**
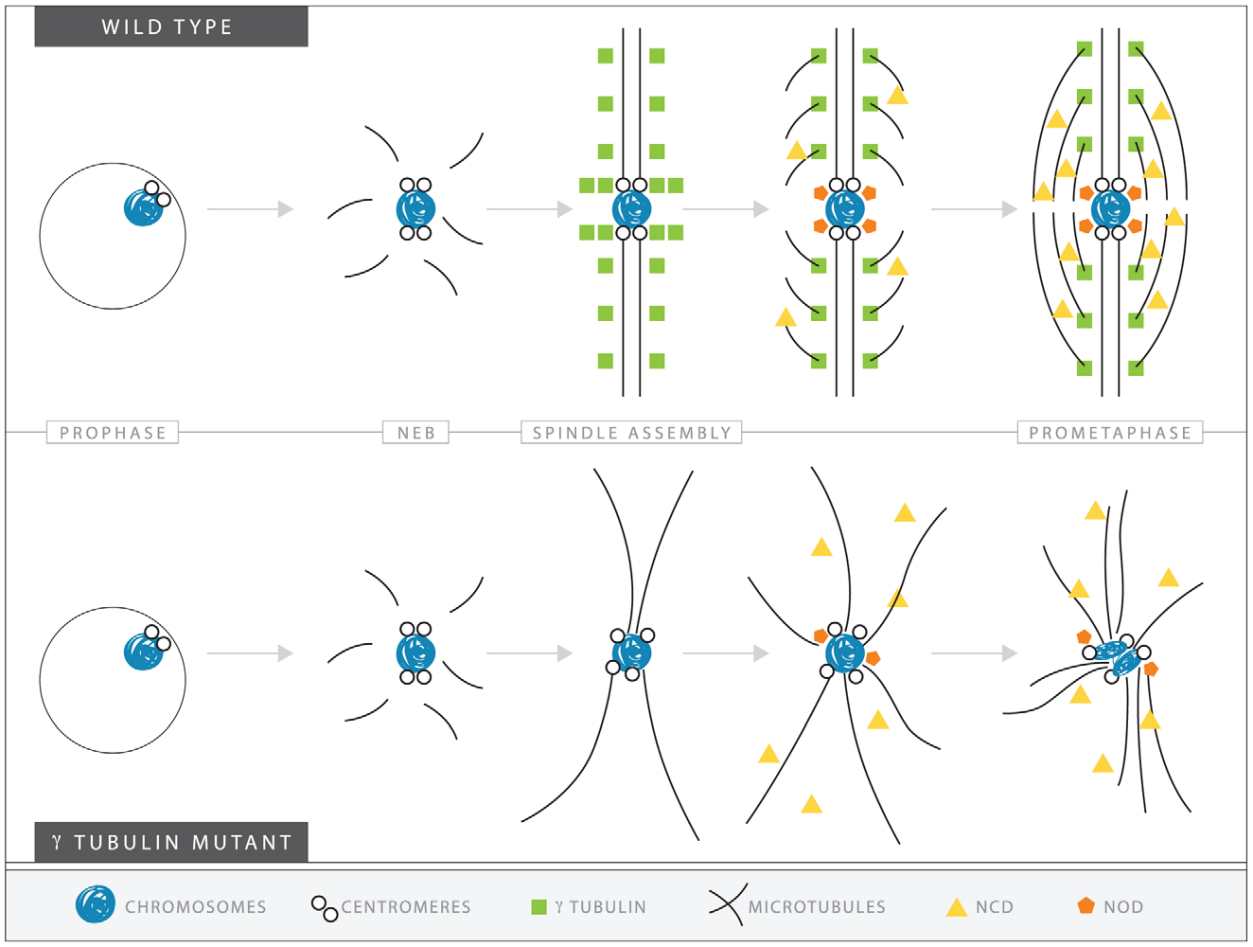
A speculative hypothesis for the functions of γTub37C in meiosis I in *Drosophila* oocytes. In wild-type oocytes (top) microtubules are recruited to the chromosomes after nuclear envelope breakdown (NEB). γTub37C complexes nucleate and stabilize additional microtubules, especially the kinetochore microtubules that attach kinetochores to the developing spindle poles. Ncd bundles the microtubules together to further develop the tapered, bipolar spindle with the help of additional factors, such as the spindle pole component, D-TACC, and the chromosome passenger complex (not shown). Nod utilizes the microtubules to push the chromosomes toward the spindle midzone. In the absence of functional γTub37C (bottom) microtubules can still be recruited to the chromosomes after NEB but nucleation of additional microtubules, particularly kinetochore microtubules, is delayed and existing microtubules are unstable. In the absence of robust microtubules the ability of Ncd to bundle microtubules into a tapered, bipolar spindle is inhibited. Nod function would also be inhibited which, in combination with disrupted kinetochore microtubule attachments, would lead to chromosome orientation and alignment defects.

Based on the data presented above, we suggest a speculative model for the role of *γ*Tub37C in meiosis I. We propose that during meiosis I in oocytes, *γ*Tub37C may be controlling spindle assembly and maintenance through several different mechanisms. As outlined in Figure 9, *γ*Tub37C localizes to the microtubules in wild-type oocytes. Loss of *γ*Tub37C would result in changes in microtubule nucleation and stability. The kinetochore microtubules required for aligning and orienting the kinetochores would be especially affected by these changes. According to our model, these defects would cause spindles to have abnormal morphology, which explains our frequent observation of extremely morphologically abnormal spindles at prometaphase I and metaphase I spindles that appear to be two thick microtubule bundles (see Figure 5B). The mislocalization of the spindle pole component D-TACC in *γtub37C* mutant oocytes would likely further disrupt spindle morphology.

Decreased kinetochore microtubules in *γtub37C* mutant oocytes would result in a failure of chromosomes to attach to opposite spindle poles and properly biorient, explaining the chromosome alignment and co-orientation defects that we have observed. These defects in spindle assembly would likely also inhibit the ability of other proteins, such as the Ncd kinesin, to bundle microtubules together, which would further impair spindle assembly and chromosome alignment [37], [38]. As predicted by such a hypothesis, we observed clear second-site noncomplementation between an allele of *ncd* and a small deficiency uncovering *γtub37C* in terms of defects in spindle structure, such as non-tapered spindle poles. Such defects were observed in 56% of these doubly heterozygous oocytes. Spindle defects were also observed in 38% of oocytes heterozygous for both *γtub37C^P162L^* and *ncd* mutations (data not shown). Spindle defects were observed in only 11% of *ncd* heterozygotes, 5% of *γtub37C^P162L^* heterozygotes, and 10% of oocytes heterozygous for the deficiency uncovering *γtub37C*. These results suggest that some of the defects we observe in *γtub37C* mutant oocytes could be partially mediated by an impairment (perhaps secondarily) in the ability of Ncd to properly function on the microtubules.

Our model also proposes that the defects created by the loss of γTub37C would also impede the functioning of Nod, a chromokinesin-like protein whose plus-end polymerization function is required for the polar ejection force [39], [40], [41]. Indeed, we can imagine that the impairment of Nod function might lead to the defects in chromatin morphology. Such a proposal is consistent with our observation that chromosomes on the most aberrant spindles typically displayed the most severe morphology defects. Abnormal microtubule bundles, impaired attachment of Nod to the chromosomes arms, and a lack of proper kinetochore microtubule attachments would lead to a cascade of defects that affect various aspects of chromosome and spindle structure.

In summary, our analysis of staged mutant oocytes and sophisticated microscopy demonstrate that *γ*Tub37C is present in the meiosis I spindle of *Drosophila* oocytes and plays important roles in spindle assembly, maintenance and positioning, as well as in chromosome positioning, orientation and morphology. Multispectral imaging allowed for detection of endogenously expressed *γ*Tub37C in the meiosis I spindle. Furthermore, a point mutation that disrupted localization caused severe spindle defects, strongly suggesting that correct localization of *γ*Tub37C to the spindle is necessary for this role. *γ*-Tubulin also appears to play an important role during meiosis in mammalian oocytes and knock down of *γ*-tubulin by siRNA in mouse oocytes leads to chromosome misalignment and changes in spindle structure [3], [5]. Our work shows that *Drosophila* can be used as a model for understanding the function of *γ*-tubulin in acentriolar bipolar spindle assembly.

## Materials and Methods

### Stocks

Flies were maintained on standard food at 25°C. Wild-type flies were *yw*; *pol*. γ*tub37C^P162L^* flies were *yw*; γ*tub37C^P162L^* and the mutation was generated by EMS mutagenesis in the Hawley laboratory (see below). γ*tub37C^3^/CyO* and *w^1118^*; *Df(2L)Exel6043, P{XP-U}Exel6043/CyO* (deficiency uncovering γ*tub37C*) were obtained from the Bloomington Stock Center and γ*tub37C^3^*/*Df* flies were created by crossing the two stocks.

### Isolation of the *γtub37C^P162L^* mutation

The γ*tub37C^P162L^* mutation was isolated in the course of a screen for EMS-induced recessive female sterile mutants. Although females homozygous for the γ*tub37C^P162L^* mutation exhibited complete sterility, homozygous males were unaffected. γ*tub37C^P162L^* homozygous females laid externally wild-type looking eggs that failed to hatch. Embryos from homozygous γ*tub37C^P162L^* mothers arrested with one or a few spindle-like structures (data not shown). The spindles were often large, wide, and had chromosomes or chromosome fragments distributed across the spindle structure rather then being aligned on the metaphase plate (data not shown).

Deficiency mapping using the sterility phenotype narrowed the location of the γ*tub37C^P162L^* mutation to region 37C-D, which includes γ*tub37C*. By sequencing the γ*tub37C* gene from γ*tub37C^P162L^* homozygous flies we identified a C to T transition at position 834 that results in a P to L change at amino acid 162 in exon 3. A complementation test using the γ*tub37C^3^* null mutation confirmed the sterility of γ*tub37C^P162L^* was due to the mutation in γ*tub37C*.

For sequencing the γ*tub37C* gene, genomic DNA was prepared from single flies by standard protocol [42]. The following gene primers were used for amplification of the γ*tub37C* gene and for sequencing: 5′ CCTACCTCGTTCAGAGTTATTT, 5′ TAATGACTTCCACTTCCATC, 5′ TGGTCTTTCGAACGCTTGTC, 5′ CCACCGCCGTGCTTGGAGAG, 5′ GACAAGCGTTCGAAAGACCA, and 5′ CTCTCCAAGCACGGCGGTGG.

### Immunostaining

Oocytes were fixed by one of two methods. For all samples except one replicate of the D-TACC prometaphase I experiments, ovaries were dissected from yeasted females in 1× Robb's media (55 mM sodium acetate, 8 mM potassium acetate, 20 mM sucrose, 2 mM glucose, 0.44 mM MgCl_2_, 0.1 mM CaCl_2_ and 20 mM HEPES, pH 7.4) containing 1% Bovine Serum Albumin (BSA). For prometaphase I-enriched preparations, females were yeasted for 2–3 days with males [14] (see Table 1 for level of enrichment). For metaphase I-enriched preparations, virgin females were yeasted for 4–5 days post-eclosion [14] (see Table 3 for level of enrichment). Ovaries were fixed using a 1× fix buffer (100 mM potassium cacodylate, 100 mM sucrose, 40 mM sodium acetate and 10 mM EGTA) and 8% formaldehyde (Ted Pella) for 4–5 minutes. After fixation oocytes were washed three times in PBS plus 0.1% triton-X-100 (PBST) and vitelline membranes were removed manually using the rough end of two frosted slides. After further washing with PBST, oocytes were blocked with 5% Normal Goat Serum (NGS) for at least one hour. Oocytes were incubated overnight in primary antibodies in PBST and 5% NGS at 4°C. After several washes with PBST, oocytes were incubated at room temperature for 4–5 hours or 4°C overnight with secondary antibodies in PBST and 5% NGS. 1.0 µg/mL 4′6-diamididino-2-phenylindole (DAPI) or 2.5 µg/mL Hoechst 34580 (Invitrogen) DNA dye was added during the last 10–20 minutes of incubation. Oocytes were washed three times in PBST and then mounted in ProLong Gold (Invitrogen).

For fixed preparation using only DAPI, ovaries were fixed under the same conditions as above and washed three times in PBST. Ovaries were teased using gentle pipetting and 2.0 µg/mL DAPI was added for 20 minutes. After three washes in PBST oocytes were mounted in ProLong Gold (Invitrogen).

To ensure the mislocalization of D-TACC was repeatable under different fixation conditions, the prometaphase I experiments with the anti-D-TACC antibody were replicated using the Buffer A protocol described in McKim et al. [43]. Females were dissected in 1× Robb's media plus 1% BSA and fixed for 10 minutes in 1× Buffer A (15 mM PIPES, pH 7.4, 80 mM KCl, 20 mM NaCl, 2 mM EDTA, 0.5 mM EGTA), 1 mM DTT, 0.5 mM spermidine, 0.15 mM spermine and 4% paraformaldehyde. Samples were washed three times in a Buffer A solution lacking formaldehyde but containing triton-X-100. Vitelline membranes were removed manually using the rough end of two frosted slides and washed three more times. Oocytes were blocked for 30 minutes in a Buffer A solution containing 10% NGS. Antibodies were spun for 10 minutes at 4°C in the same solution and then added to oocytes overnight at 4°C. Oocytes were washed in a Buffer A solution containing 0.2% BSA and then incubated with secondary antibodies in a Buffer A solution with 10% NGS for 3–5 hours. DAPI was added for 10–15 minutes and samples were washed in a Buffer A solution before mounting in ProLong Gold.

The primary antibodies were used at the following concentrations: rat anti-α-tubulin (AbD Serotec, NC 1∶250), mouse anti-α-tubulin DM1a (Sigma-Aldrich 1∶100), rabbit anti-γtub37C ([26] 1∶100), rabbit anti-D-TACC ([44] 1∶250), rat anti-CID ([45] 1∶1000) and rabbit anti-phosphorylated-histone H3 at serine 10 (Millipore 1∶500 or 1∶250). Secondary Alexa-488 or Alexa-555 conjugated antibodies (Molecular Probes) were used at a dilution of 1∶400.

FISH was performed as described by Xiang and Hawley [46], with the following modifications. Incubation and hybridization temperature was 30°C and annealing temperature was 91°C. Part of the 359-bp repeat on the *X* chromosome conjugated to Alexa Fluor 488 and the AATAT repeat primarily on the *4^th^* chromosome conjugated to Cy3 were chosen as probes as previously described [47], [48].

For fixed experiments not requiring spectral unmixing, the DeltaVision microscopy system was used (Applied Precision, Issaquah, WA). The system is equipped with an Olympus 1×70 inverted microscope and high-resolution CCD camera. The images were deconvolved using the SoftWoRx v.25 software (Applied Precision).

For spectral unmixing experiments images were acquired with an LSM-710 confocal laser scanning microscope (Carl Zeiss Microimaging, Inc., Jena, Germany) using either a 40× 1.3 NA Plan-Neofluar or a 40× 1.3 NA Plan-Apochromat oil objective. Images were collected using a pinhole of one airy unit and a pixel dwell time of 1.6 µs. Each line was averaged eight times in the acquisition. Z stacks were obtained at 0.5 µm intervals. All antibody imaging was performed using the spectral detection channel of the microscope with the MBS 488/561 excitation dichroic with 9.8 nm resolution and collecting from 494 to 660 nm. All focusing and zooming operations were performed with excitation at 561 nm only (for visualization of the Alexa Fluor 555 α-tubulin staining) to avoid photobleaching of the weak Alexa Fluor 488 (AF488) staining of γ-tubulin. All confocal imaging was performed with 488 nm excitation only to maximize the AF488 signal relative to AF555, as we found that 488 nm excitation was sufficient to observe the α-tubulin-AF555 signal. Reference spectra for secondary antibodies were obtained every day that imaging was performed under identical imaging conditions except with small gain and laser power changes. Variation in reference spectra between the two objectives used from day to day was negligible. The reference spectra were obtained using the secondary antibodies mounted in ProLong Gold (identical to biological sample mounting). Hoechst or DAPI imaging was accomplished with 405 nm excitation and a MBS 405 excitation dichroic and a 415–480 bandwidth collection channel. Hoechst or DAPI imaging was done under identical zoom and Z stack settings to the visible light imaging for each sample.

All image processing was accomplished using ImageJ functionality as well as custom-written plugins for binning and spectral unmixing. Spectral images were linear unmixed using standard linear least squares algorithms. Residuals images were generated for each wavelength at each Z position. These residuals were carefully inspected, with close attention paid to channels containing AF488 to ensure that signal from those channels conformed to the expected spectrum for AF488. In all images, these residuals were completely random in the AF488 channels and showed minimal variation in AF555 channels. Maximum projections of selected slices containing spindles were performed for presentation purposes for both the unmixed images as well as the Hoechst or DAPI images. Maximum projection of the entire collected Z stack was avoided due to non-specific staining above and below the spindle.

### Live-imaging

Live-imaging was performed as describes in Hughes et al. [15]. Briefly: approximately stage 13 oocytes were dissected from ovaries of 2–3 day-old, well-fed adult females and the oocytes were aligned in halocarbon oil 700 (Sigma) in a well made on a no. 1 ½ coverslip. Oocytes were injected using standard micro-injection procedures with an approximately 1∶1 ratio of bovine or porcine rhodamine-conjugated tubulin minus glycerol (Cytoskeleton) and Quant-iT OliGreen ssDNA Reagent (Invitrogen) diluted 0.7 fold with water. After injection, oocytes were covered with a piece of YSI membrane.

The well slides were placed on a temperature-controlled bionomic controller (Technology, Inc) set at 23.5°C. Oocytes were imaged using an LSM-510 META confocal microscope (Zeiss) with a Plan-APO 40× objective (1.3 NA) with a zoom of 2–2.5 or an alpha plan-fluar 100× (1.4 NA) with a 1.5 zoom. Images were acquired using the AIM software v4.2 by taking a 10 series Z stack at 1 micron intervals with 20 seconds between acquisitions which resulted in a set of images approximately every 45 seconds. Images were transformed into 2D projections and concatenated into videos using the AIM software v4.2.

### Western blot analysis

For each genotype, 50 pairs of ovaries from 2–3 day-old, yeast-fed females were dissected in 1× PBS and homogenized in 50 µL of cold lysis buffer containing 150 mM NaCl, 50 mM Tris (pH 6.8), 2.5 mM EDTA, 2.5 mM EGTA, 0.1% Triton-X, and protease inhibitor cocktail (Sigma-Aldrich). Ovary lysates were cleared by centrifugation twice at 14,000 rpm for 15 minutes at 4°C. Equivalent volumes of ovary lysates per genotype were combined with 2× SDS sample buffer, boiled for five minutes, and the solubilized proteins were analyzed by Western blot using standard techniques. The primary antibody used for Western blot was rabbit anti-DrosC γTub37C at a dilution of 1∶500. Immunoreactivity was detected using an alkaline phosphatase-conjugated rabbit secondary antibody (Jackson ImmunoResearch) and the nitroblue tetrazolium and 5-bromo-4-chloro-3-indolyl phosphatase (NBT/BCIP, Invitrogen) reagents.

## Supporting Information

Figure S1 Mutations in *γtub37C* cause spindle and chromosome defects in most, but not all, prometaphase I oocytes. Fixed oocytes were treated with antibodies against α-tubulin and histone H3 phosphorylated at serine 10 (phH3S10), as well as the DNA dye DAPI. Arrowheads point to aberrant phH3S10-positive threads projecting from the chromosome mass. (A) A *γtub37C^P162L^* mutant oocyte with a spindle lacking directionality and chromosomes showing morphology and alignment defects. (B) A *γtub37C* ^3^ */Df* mutant oocyte with chromosomes and microtubules failing to show clear orientation. (C) A *γtub37C^P162L^* mutant oocyte with a tapered bipolar spindle. (D) A *γtub37C^P162L^* mutant oocyte with a long, thin, barrel-like spindle. (C) and (D) are projections of Z stacks while (A) and (B) are single plane images from the Z stack to highlight the phH3S10 threads. Scale bars are in microns.
(TIF)

Figure S2 The phH3S10 antibody robustly localizes to DNA threads connecting achiasmate chromosomes. Fixed *FM7w/yw*; *pol* oocytes were treated with antibodies against α-tubulin and histone H3 phosphorylated at serine 10 (phH3S10), as well as the DNA dye DAPI. Heterozygosity for the balancer chromosome *FM7w* results in oocytes with achiasmate *X*s, as well as achiasmate *4^th^* chromosomes. The phH3S10 antibody localizes to the DNA threads connecting both sets of achiasmate chromosomes. Arrowheads highlight a few of the threads. Single plane images are shown in order to highlight the phH3S10 fluorescing threads. Scale bars are in microns.
(TIF)

Figure S3 γTub37C protein is expressed in *γtub37C^P162L^* mutant ovaries. Shown is a Western blot using the DrosC anti-γTub37C antibody recognizing the C-terminus of γTub37C. Each lane represents lysate from 50 ovaries. Lanes 1 and 2 were loaded with 1 and 2 µl, respectively, of lysate from wild-type ovaries. Lanes 3 and 4 were loaded with 1 and 2 µl, respectively, of lysate from *γtub37C^P162L^* mutant ovaries. Lanes 5–8 were loaded with two independent samples from *γtub37C^3^/Df* mutant ovaries with 1 (lanes 5 and 7) or 2 (lanes 6 and 8) µl of lysate. Lanes marked M show the Precision Plus Protein All Blue standard and unlabeled lanes were not directly loaded with sample. Indicated is the expected approximately 50 kDa band for γTub37C seen in wild-type and *γtub37C^P162L^* mutant ovaries that is absent in *γtub37C^3^/Df* mutant ovaries.
(JPG)

Table S1 Orientation of CID foci.
(PDF)

Table S2 Position of the oocyte nucleus within the oocyte.
(PDF)

Video S1 The spindle and chromosomes remain relatively stable for long periods in a wild-type oocyte. OliGreen (yellow) labels the DNA and rhodamine-conjugated tubulin (blue) labels the spindle. The video is a projection from Z stack.
(MOV)

Video S2 The spindle loses and regains bipolarity after nuclear envelope breakdown in a living *γtub37C^P162L^* mutant oocyte. OliGreen (yellow) labels the DNA and rhodamine-conjugated tubulin (blue) labels the spindle. The video is a projection from Z stack.
(MOV)

Video S3 Defects in spindle morphology, spindle position, and chromosome alignment are observed in a living *γtub37C^P162L^* mutant oocyte. Stills from Video S3 are shown in Figure 4. OliGreen (yellow) labels the DNA and rhodamine-conjugated tubulin (blue) labels the spindle. The video is a projection from Z stack.
(MOV)

Video S4 The spindle and microtubules move rapidly resulting in the dissolution of the spindle in a living *γtub37C^P162L^* mutant oocyte. OliGreen (yellow) labels the DNA and rhodamine-conjugated tubulin (blue) labels the spindle. Videos are projections from Z stack.
(MOV)
