# Supplementary figures and images for: Gamma-Tubulin Is Required for Bipolar Spindle Assembly and for Proper Kinetochore Microtubule Attachments during Prometaphase I in Drosophila Oocytes
Source: PLoS Genet. 2011 Aug 11;7(8):e1002209. doi: 10.1371/journal.pgen.1002209 (PMC3154956; doi:10.1371/journal.pgen.1002209)

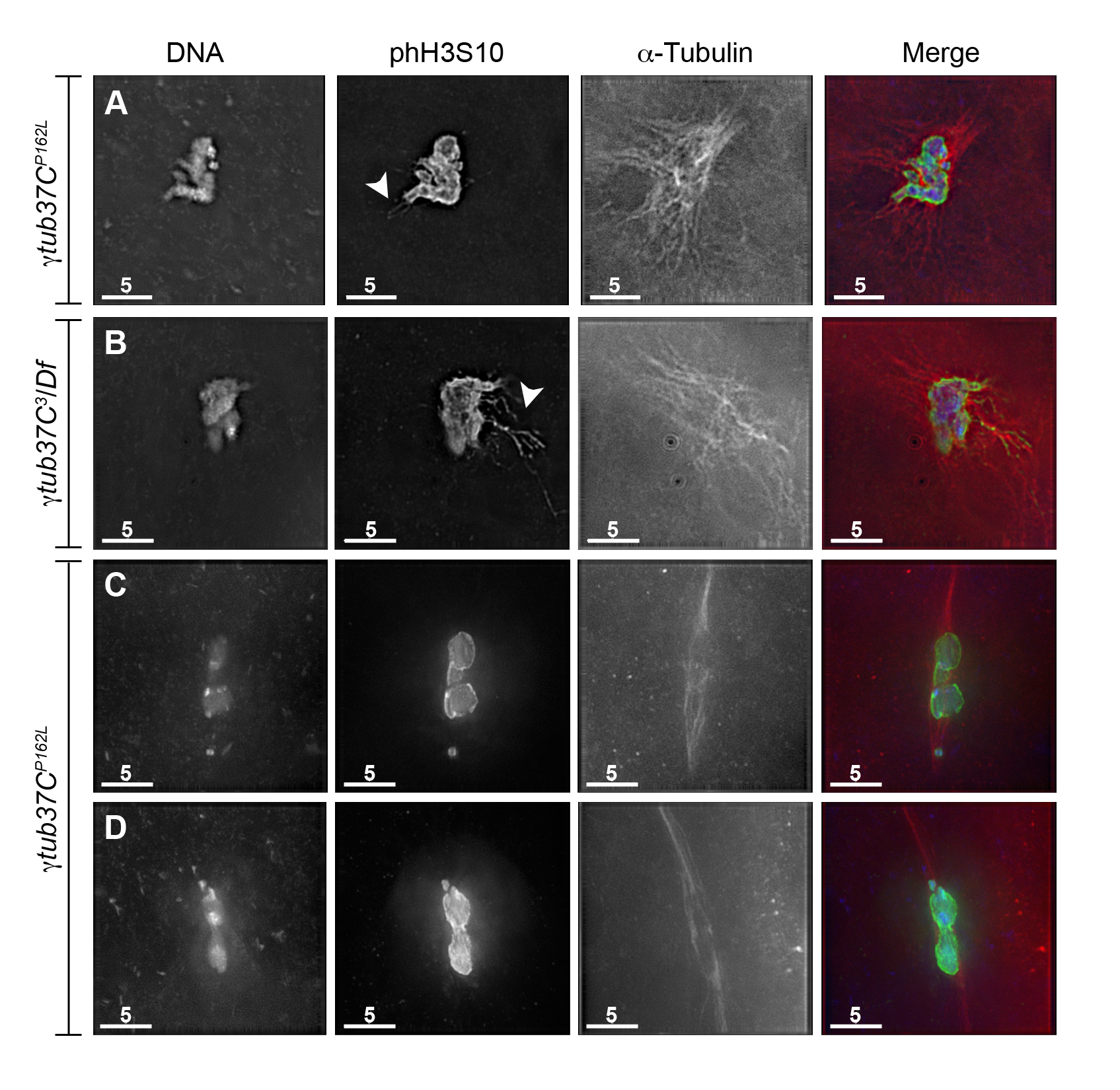

Supplement: Figure S1 — Mutations in γtub37C cause spindle and chromosome defects in most, but not all, prometaphase I oocytes. Fixed oocytes were treated with antibodies against α-tubulin and histone H3 phosphorylated at serine 10 (phH3S10), as well as the DNA dye DAPI. Arrowheads point to aberrant phH3S10-positive threads projecting from the chromosome mass. (A) A γtub37CP162L mutant oocyte with a spindle lacking directionality and chromosomes showing morphology and alignment defects. (B) A γtub37C 3 /Df mutant oocyte with chromosomes and microtubules failing to show clear orientation. (C) A γtub37CP162L mutant oocyte with a tapered bipolar spindle. (D) A γtub37CP162L mutant oocyte with a long, thin, barrel-like spindle. (C) and (D) are projections of Z stacks while (A) and (B) are single plane images from the Z stack to highlight the phH3S10 threads. Scale bars are in microns. (TIF) [file pgen.1002209.s001.tif]

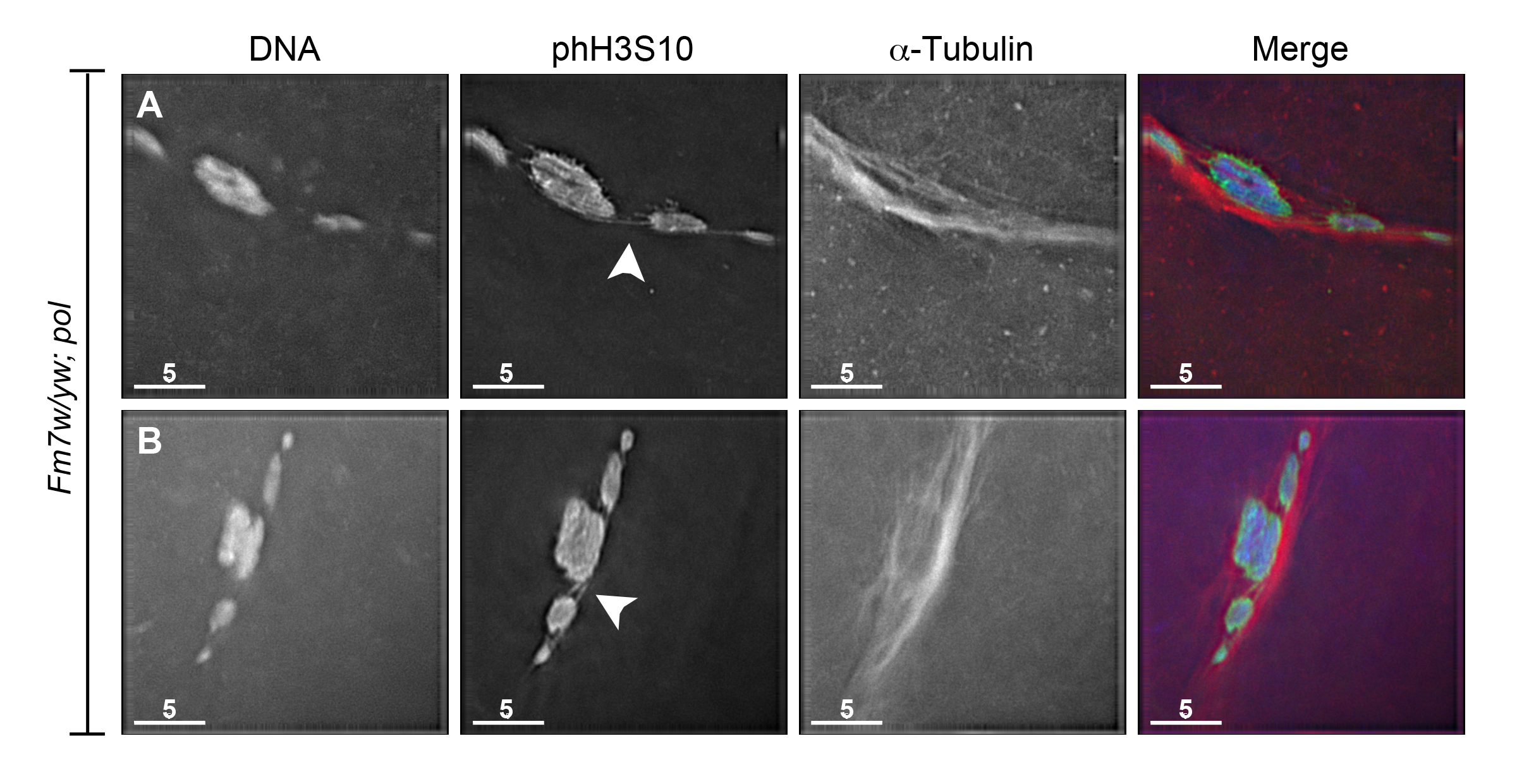

Supplement: Figure S2 — The phH3S10 antibody robustly localizes to DNA threads connecting achiasmate chromosomes. Fixed FM7w/yw; pol oocytes were treated with antibodies against α-tubulin and histone H3 phosphorylated at serine 10 (phH3S10), as well as the DNA dye DAPI. Heterozygosity for the balancer chromosome FM7w results in oocytes with achiasmate Xs, as well as achiasmate 4th chromosomes. The phH3S10 antibody localizes to the DNA threads connecting both sets of achiasmate chromosomes. Arrowheads highlight a few of the threads. Single plane images are shown in order to highlight the phH3S10 fluorescing threads. Scale bars are in microns. (TIF) [file pgen.1002209.s002.tif]

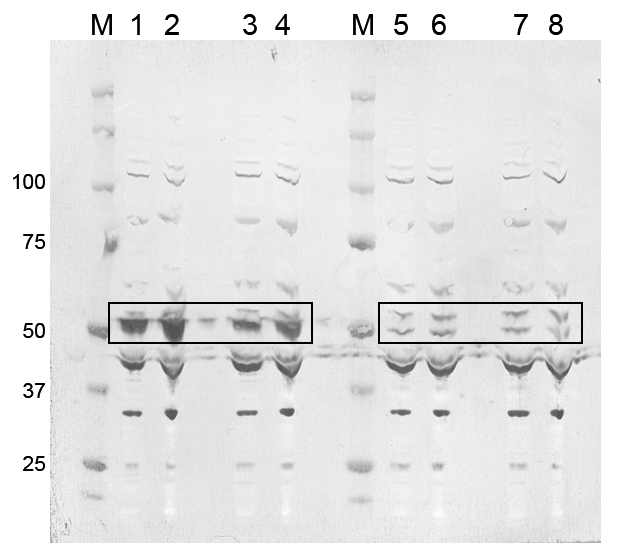

Supplement: Figure S3 — γTub37C protein is expressed in γtub37CP162L mutant ovaries. Shown is a Western blot using the DrosC anti-γTub37C antibody recognizing the C-terminus of γTub37C. Each lane represents lysate from 50 ovaries. Lanes 1 and 2 were loaded with 1 and 2 µl, respectively, of lysate from wild-type ovaries. Lanes 3 and 4 were loaded with 1 and 2 µl, respectively, of lysate from γtub37CP162L mutant ovaries. Lanes 5–8 were loaded with two independent samples from γtub37C3/Df mutant ovaries with 1 (lanes 5 and 7) or 2 (lanes 6 and 8) µl of lysate. Lanes marked M show the Precision Plus Protein All Blue standard and unlabeled lanes were not directly loaded with sample. Indicated is the expected approximately 50 kDa band for γTub37C seen in wild-type and γtub37CP162L mutant ovaries that is absent in γtub37C3/Df mutant ovaries. (JPG) [file pgen.1002209.s003.jpg]
