# Supplementary material for: Gamma-Tubulin Is Required for Bipolar Spindle Assembly and for Proper Kinetochore Microtubule Attachments during Prometaphase I in Drosophila Oocytes
Source: PLoS Genet. 2011 Aug 11;7(8):e1002209. doi: 10.1371/journal.pgen.1002209 (PMC3154956; doi:10.1371/journal.pgen.1002209)
Supplement: Table S1 — Orientation of CID foci. (PDF) [file pgen.1002209.s004.pdf]

TABLE S1

ORIENTATION OF CID FOCI

GENOTYPE	BI-ORIENTED[1]	MALORIENTED[2]	MONO-ORIENTED[3]	CLUSTERED[4]	MORE THAN EIGHT FOCI[5]	TOTAL
+/+	26	2	0	0	0	28
γtub37C^{P162L}	13	19	0	0	2	34
γtub37C^{3}*/Df*	6	18	4	2	4	34

1. Four CID foci are pointing in opposite directions.
2. More than four CID foci are pointed in one direction or CID foci are oriented in more than two directions.
3. All CID foci are oriented towards the same direction.
4. CID foci are clustered close together.
5. More than 8 CID foci are present indicating loss of sister chromatid cohesion.
