# Supplementary material for: Gamma-Tubulin Is Required for Bipolar Spindle Assembly and for Proper Kinetochore Microtubule Attachments during Prometaphase I in Drosophila Oocytes
Source: PLoS Genet. 2011 Aug 11;7(8):e1002209. doi: 10.1371/journal.pgen.1002209 (PMC3154956; doi:10.1371/journal.pgen.1002209)
Supplement: Table S2 — Position of the oocyte nucleus within the oocyte. (PDF) [file pgen.1002209.s005.pdf]

TABLE S2

POSITION OF THE OOCYTE NUCLEUS WITHIN THE OOCYTE

	GENOTYPE	ANTERIOR	MIDDLE	POSTERIOR	TOTAL
STAGE 11-12	+/+	50	1	0	51
	γtub37C^{P162L}	33	8	2	43
	γtub37C^{3}/Df	22	9	3	34
STAGE 13-14	+/+	40	0	0	40
	γtub37C^{P162L}	24	14	3	41
	γtub37C^{3}/Df	17	20	9	46
